# Healthcare access, satisfaction, and health-related quality of life among children and adults with rare diseases

**DOI:** 10.1186/s13023-022-02343-4

**Published:** 2022-05-12

**Authors:** Kathleen Bogart, Amanda Hemmesch, Erica Barnes, Thomas Blissenbach, Arthur Beisang, Patti Engel, The Chloe Barnes Advisory Council on Rare Diseases, Jakub Tolar, Tim Schacker, Lisa Schimmenti, Nicole Brown, Kelly Morrison, Tony Albright, Matt Klein, Julia Coleman, Karl Nelsen, Rae Blaylark, Karri LaFond, Sheldon Berkowitz, Kris Ann Schultz, Kerry Hansen, Soraya Beiraghi, Barbara Joers, David Tilstra, Amy Gaviglio, Lee A. Jones, Abigail Miller, Jackie Foster

**Affiliations:** 1 School of Psychological Science, Oregon State University, Corvallis, OR USA; 2 Department of Psychology, St. Cloud State University, St. Cloud, MN USA; 3 Chloe Barnes Advisory Council on Rare Diseases, Minneapolis, MN USA; 4 Medical School, University of Minnesota, 420 Delaware St SE, Minneapolis, MN 55455 USA; 5 Gillette Children’s Specialty Healthcare, St. Paul, MN USA; 6 Engage Health Inc., Eagan, MN USA; 7 University of Minnesota, Minneapolis, MN USA; 8 4ES Corporation, San Antonio, TX USA; 9 Minnesota House of Representatives, St. Paul, MN USA; 10 Minnesota State Senate, Mayo Clinic, St. Paul, MN USA; 11 Minnesota State Senate, Edina, MN USA; 12 Fairview Southdale Hospital and Fairview Ridges Hospital, Edina, MN USA; 13 Sickle Cell Foundation of Minnesota, Minneapolis, MN USA; 14 Minneapolis, MN USA; 15 Children’s Minnesota, Minneapolis, MN USA; 16 Fairview, Minneapolis, MN USA; 17 CentraCare, St. Cloud, MN USA; 18 Rebiotix Inc., Roseville, MN USA; 19 PreferredOne®, Golden Valley, MN USA; 20 Be the Match, Minneapolis, MN USA

**Keywords:** Rare disease, Stigma, Depression, Anxiety, Patient satisfaction, Health-related quality of life

## Abstract

**Background:**

Research in a variety of countries indicates that healthcare access and health-related quality of life are challenged among people with a variety of rare diseases (RDs). However, there has been little systematic research on the experiences of children and adults with RDs in the American healthcare system that identifies commonalities across RDs. This research aimed to: (1) Describe demographics, disease characteristics, diagnostic experiences, access to healthcare, knowledge about RDs, support from healthcare professionals, and patient satisfaction among people with RDs and their caregivers; (2) examine predictors of patient satisfaction among adults with RDs; (3) compare health-related quality of life and stigma to US population norms; 4) examine predictors of anxiety and depression among adults and children with RDs.

**Results:**

This large-scale survey included (*n* = 1128) adults with RD or parents or caregivers of children with RDs representing 344 different RDs. About one third of participants waited four or more years for a diagnosis and misdiagnosis was common. A subset of participants reported experiencing insurance-related delays or denials for tests, treatments, specialists, or services. Approximately half of participants felt their medical and social support was sufficient, yet less than a third had sufficient dental and psychological support. Patients were generally neither satisfied or dissatisfied with their healthcare providers. Major predictors of satisfaction were lower stigma, lower anxiety, shorter diagnostic odyssey, greater physical function, and less pain interference. Adults and children with RDs had significantly poorer health-related quality of life and stigma in all domains compared to US norms. Predictors of both anxiety and depression were greater stigma/poor peer relationships, fatigue, sleep disturbance, limited ability to participate in social roles, and unstable disease course.

**Conclusions:**

People in the U.S. with RDs have poor health-related quality of life and high stigma. These factors are related to patient satisfaction and healthcare access, including diagnostic delays and misdiagnosis. Advocacy work is needed in order to improve healthcare access and ultimately health-related quality of life for children and adults with RDs.

**Supplementary Information:**

The online version contains supplementary material available at 10.1186/s13023-022-02343-4.

## Background

Diseases are characterized as rare in the United States when their prevalence is less than 200,000 cases. Although each rare disease or disorder (RD) affects a small number, together there are more than 7000 different RDs affecting approximately 1 in 10 Americans [1]. Despite etiological differences, there are commonalities across diverse RDs. Most RDs are chronic, genetic, involve multiple body systems and medical specialties, and few have effective treatments or cures [2–4]. Given these commonalities, examining factors like healthcare access and satisfaction, and health-related quality of life among many different RDs collectively can provide insight into how to address these challenges through broad healthcare policy and psychosocial support.

### Healthcare access

RDs are challenging to diagnose, treat, and self-manage, and healthcare coverage for treatments is inconsistent [5]. A number of challenges have been acknowledged in the scientific literature, yet most research has been limited by small sample sizes or by focusing on specific RDs or clinics. In a Dutch study of adults with rare sarcomas, Drabbe et al. [6] found that most participants felt they had insufficient guidance on treatments. In a survey of U.S. adults with primary mitochondrial disease, the most significant impediment to healthcare was that more than half of participants indicated their primary care provider did not sufficiently understand their condition [7]. The majority of this sample saw three or more specialists [7]. Yet, more than a quarter did not have a specialist in their RD, or their specialists were a great distance away. Most reported having been sometimes or frequently given confusing or contradictory info about healthcare treatments. An Australian study of adults with RDs found high levels of diagnostic delay, two thirds had seen at least three doctors to receive a diagnosis, nearly half had received at least one previous misdiagnosis, and three quarters received insufficient information at the time of diagnosis [5]. Regarding support, two thirds of participants agreed they had sufficient medical support, while one third or less agreed they received sufficient social, financial, and psychological support [5]. In our current study, we report on a large U.S. sample of people with a variety of RDs.

### Patient satisfaction

Patient satisfaction—the extent to which patients believe their healthcare is competent, accessible, and effective [8]—is a common outcome of interest because it is linked to patient knowledge, self-management, and adherence [8]. In research on more prevalent conditions, physical functioning, symptom improvement, older age, lower anxiety and depression, having received explanation of likely cause and duration of condition, and no unmet expectations were associated with greater patient satisfaction [9]. Little work has examined patient satisfaction in the RD space. In the RD population, an Australian study assessed overall satisfaction with adult and pediatric health services with single item measures, finding that one third of participants were satisfied or very satisfied with adult services, and half of participants were satisfied or very satisfied with pediatric services [5]. The current study will address a gap in the research by examining factors associated with patient satisfaction in a large and diverse sample of RDs using a validated patient satisfaction scale for the first time. Based on the broader RD literature, we predicted that factors such as diagnostic delay, disease characteristics, anxiety, depression, and stigma would be associated with patient satisfaction.

### Health-related quality of life

The challenges involved with living with a RD described above have been found to take a toll on health-related quality of life—an individual’s perceived physical, mental, and social health [10]. Anxiety and depression are particularly of interest as outcome measures because they are psychological conditions that cross-cut etiologies and have a variety of effective treatments (e.g., psychotherapy, medications) that can be used even if RD treatments do not exist. A large cross-RD study found that adults with RDs had poorer health-related quality of life—including anxiety, depression, fatigue, pain interference, physical function, and ability to participate in social roles and activities—compared to general population norms and to norms of people living with chronic but prevalent conditions [11]. Of particular note, anxiety and depression were associated with younger age, female gender, more years since diagnosis, lower income, fatigue, pain interference, and lower physical function. People with RDs also experience structurally enacted, interpersonally enacted, and felt stigma [12]. Structurally enacted stigma describes when people with RDs experience invalidation and disbelief by healthcare practitioners and an overall lack of accessibility in terms of workplace accommodations and social participation. Interpersonally, people with RDs experience a lack of understanding or recognition from others and insufficient social support. Some internalize this stigma and feel shame or pressure to hide their illness from others. Less work has examined health-related quality of life among children with RDs, but some studies of specific conditions have found poor pediatric quality of life [13, 14]. Based on the broader RD literature, we predict that anxiety and depression symptoms in adults and children will be associated with demographic characteristics such as age, and time since diagnosis, disease characteristics such as fatigue, pain, lower physical function, and peer acceptance or stigma.

### Current study

The purpose of the study is to:

1. Describe demographics, disease characteristics, diagnostic experiences, access to healthcare, knowledge about RDs, support from healthcare professionals, and patient satisfaction among people with RDs and parents/caregivers to people with RDs.
2. Compare health-related quality of life and stigma to U.S. norms.
3. Examine predictors of patient satisfaction among adults with RDs.
4. Examine predictors of anxiety and depression among adults and children with RDs.

## Method

### Participants

For this online study, we recruited adults living with a RD or parents/caregivers to someone living with a RD. Participants who recovered or parents/caregivers to individuals who died were excluded from this study to maintain a focus on current experiences with healthcare access and health-related quality of life. Participants were recruited from throughout the United States via various channels, including hospitals, healthcare providers, RD support organizations, social media posts, and snowball sampling (e.g., participants could invite others with RDs to complete the study). In total, 1639 individuals responded to the online survey. As part of data cleaning, we removed data from participants who did not click ‘yes’ to agree to the consent form (*n* = 32), reported they were under 18 years of age (*n* = 4), or that the person with the RD recovered or died (*n* = 116). Additionally, participants were removed from the final dataset if they did not self-report at least one RD (*n* = 317). Multiple responses submitted from the same IP addresses were checked for duplicate entries; the earliest or most complete submission was retained and any later or less complete duplicate submissions were removed. This resulted in a final sample of 1128 participants with verified self-reported RDs.

### Measures

The survey was designed to assess experiences with healthcare and health-related quality of life and is described below. The survey is available as Additional file 1.

#### Demographics

Participants were asked standard demographic questions (e.g., age, gender, race/ethnicity, marital status, highest level of education completed, annual household income, and work status). Participants were also asked if they were an adult living with a RD, if they were a parent of a child with a RD, a relative or caregiver of a person with a RD, or a paid support worker. Finally, participants were asked which state they lived in and if they lived in a large city (> 500,000 residents), medium city (100,000–500,000 residents), small town or city (< 100,000 residents), or if they lived in a rural area (outside of a town or city).

#### Rare disease diagnosis and health

Participants were asked how many RDs they/their loved one had and for the name(s) of the RDs. Next was a series of questions about time to diagnosis and experiences during the diagnostic odyssey from Molster et al. [5]. Participants were asked a series of questions, from Engel and colleagues [15], to rate their initial provider and the provider who diagnosed them about the providers’ knowledge of RDs and willingness to seek information (e.g. research databases and investigate cause of symptoms) and support from local, regional, and national experts to arrive at a diagnosis.

#### Health insurance

We developed single items asking about the type(s) of health insurance participants had, if any, and how much money they paid out-of-pocket for health claims. We also developed questions asking if participants were able to get the following easily through their insurance, or if they experienced delays or denials: diagnostic tests, FDA-approved medications, devices/medical equipment, services, investigational treatments, and off-label treatments. Finally, there were four items asking about denials related to referrals to specialists (generally and when out-of-network), medical/dental treatments when there is no available treatment for their RDs, and medical/dental treatments when there is no standard of care.

#### Knowledge and support

Participants were asked to rate their knowledge about their RD on a scale from 1–10, with 1 representing no knowledge and 10 representing complete knowledge. Based on Molster et al. [5], participants were also asked to rate how strongly they agreed or disagreed that they have received sufficient information and care from a variety of health care professionals (e.g., medical doctors, allied health professionals, patient organizations). Participants also rated how strongly they agreed that they received sufficient support in the following domains: medical, dental, social, financial, and psychological.

Items from Molster and colleagues [5] were used to measure participants’ knowledge of and access to specialist centers and specialist doctors, care coordinators, telehealth, pediatric services, and the transition from pediatric to adult services (if applicable). Two additional items were designed for this study to determine if participants relocated within or outside their state to access health care for their RD.

#### Patient satisfaction questionnaire short form

Participants also rated their overall experience with healthcare providers using the Patient Satisfaction Short Form [16]. This scale examines seven domains: general satisfaction (current sample Cronbach’s α = 0.80), technical quality (α = 0.81), interpersonal manner (α = 0.71), communication (α = 0.61), financial aspects (α = 0.73), time spent with the provider (α = 0.80), and accessibility and convenience (α = 0.75). Scores range from 1 (strongly disagree) to 5 (strongly agree), with higher numbers indicating greater satisfaction.

#### PROMIS measures

The Patient-Reported Outcomes Measurement Information System (PROMIS) was administered to assess health-related quality of life [17, 18]. For adults, PROMIS-29 includes domains of physical function (current sample Cronbach’s α = 0.94), anxiety (α = 0.91), depression (α = 0.93), fatigue (α = 0.96), sleep disturbance (α = 0.81), ability to participate in social roles and activities (α = 0.94), and pain interference (α = 0.97). For children, PROMIS-25 Pediatric Proxy includes domains of anxiety (current sample Cronbach’s α = 0.92), depression (α = 0.89), fatigue (α = 0.94), mobility (α = 0.88), pain interference (α = 0.95), and peer relationships (α = 0.95). Participants are prompted to report on experiences within the past 7 days. Each scale was normed to the general United States population such that higher scores indicate greater amounts of the domain. PROMIS norms for people with one or more common chronic diseases have also been published for the following scales (adult sample only): anxiety, depression, and fatigue [19].

#### Stigma

Enacted and internalized stigma were assessed in adults using the Neuro-QOL 8-item stigma scale [20]. The scale has been validated for use among individuals with a wide range of chronic health conditions (current sample Cronbach’s α = 0.88). Participants rated each item on a scale ranging from 1 (*never*) to 5 (*always*). Participants were prompted to report on how they were feeling “lately.” This scale was scored using published T scores calibrated such that a mean of 50 and standard deviation of 10 are representative of the norms for populations with common chronic illnesses [20]. Higher scores indicate greater perceptions of disability-related stigma.

### Procedure

The online survey platform Qualtrics was used for data collection. Potential participants were presented with informed consent information on the first page of the survey. Implied consent/assent was recorded by participants clicking ‘yes’ that they understood the nature of the study and agreed to participate before proceeding to the survey itself.

Data collection occurred from October 2020 through February 2021, during the COVID-19 pandemic. Because we were interested in establishing a baseline understanding of healthcare access, participants were asked to recall their experiences with healthcare before the pandemic (items about healthcare access, insurance, patient satisfaction). We maintained the prompts published for the PROMIS measures and stigma scale, which ask participants to report about the last 7 days, or “lately”, respectively.

Participants were invited to complete an online survey about their experiences with healthcare and their overall quality of life. If participants preferred to complete the survey on paper, they could request a hard copy with a self-addressed stamped envelope. Six paper copies were requested and the final data set included data from four paper surveys (one was returned after the recruitment deadline and the other was not returned). Minnesotans were intentionally oversampled to facilitate comparisons between them and participants living in the rest of the U.S. for a separate project [21].^1^ Participants clicked a link or scanned a QR code to access the survey. The survey took approximately 30 min to complete. Participants received no compensation for their participation.

### Data analysis

Analyses were completed using SPSS versions 28 and 25. Demographic and healthcare experience questions were presented as frequencies (see Table 1). Health-related quality of life scores and stigma scores were compared to available population and common chronic disease norms using one sample t-tests.

**Table 1:** Demographics of the RD sample

	M (SD; range)	Frequency (%)
Age of person with RD	40.90 years (21.82; 1–90)	
RD status		
Adult with RD		787 (70%)
Relative or caregiver of a person with a RD		76 (7%)
Parent of a child with a RD		252 (22%)
Other (e.g., friend; both a person with a RD and a parent to child(ren) with RD)		11 (1%)
Gender of person with RD		
Female		752 (67%)
Male		365 (32%)
Something else		10 (1%)
Race of person with RD		
American Indian or Alaska Native		9 (< 1%)
Asian or Asian American		20 (2%)
Black or African American		19 (2%)
Hispanic or Latinx		28 (3%)
Middle Eastern		6 (< 1%)
Mixed Race		15 (1%)
Native Hawaiian or Pacific Islander		2 (< 1%)
White or Caucasian		1007 (89%)
Other		16 (1%)
Education of person with RD		
Some high school or less		99 (10%)
High school diploma or GED		81 (8%)
Some college		149 (15%)
Associate’s degree technical degree		124 (13%)
Bachelor’s degree		221 (23%)
Some graduate school		59 (6%)
Graduate degree		236 (24%)
Household income of person with RD		
Under $20,000		157 (16%)
$20,000–39,999		139 (14%)
$40,000–59,999		122 (12%)
$60,000–79,999		106 (10%)
$80,000–99,999		112 (11%)
Over $100,000		253 (25%)
Number of RDs		
1		995 (88%)
2		98 (9%)
3		25 (2%)
4		6 (< 1%)
5		4 (< 1%)
RD course		
Stable		304 (34%)
Progressive		399 (44%)
Episodic		199 (22%)
Improving		6 (< 1%)
Diagnostic delay		
0–6 months		293 (30%)
7–11 months		82 (9%)
1–3 years		224 (23%)
4–6 years		106 (11%)
7–9 years		54 (6%)
10 + years		158 (16%)
Still undiagnosed		27 (3%)
Number of doctors seen to get a diagnosis		
1		152 (16%)
2–3		360 (38%)
4–5		231 (24%)
6–10		123 (13%)
11–15		31 (3%)
More than 15		43 (5%)

In preparation for conducting regressions analyses predicting patient satisfaction, anxiety, and depression, we examined whether our variables met statistical assumptions. This included checking for linearity and unusual cases via scatterplots. The assumption of independence of errors was measured with the Durbin-Watson test. We checked residuals for linearity, homoscedasticity, and independence by inspecting scatterplots and graphs of predicted vs. observed residuals. Histograms and p-p plots of standardized residuals were examined for normality. Multicollinearity was assessed with variance inflation factor and tolerance tests. If assumptions were not met, bootstrapping based on 1000 samples and percentile confidence intervals was used, a method that is robust to those violations.

Based on our literature review, we anticipated that demographics (i.e. gender, race/ethnicity, income, and age), disease characteristics (i.e. number of RDs, diagnostic delay, disease course, PROMIS sleep disturbance, PROMIS physical function, PROMIS pain interference, PROMIS fatigue, and PROMIS ability to participate in social roles and activities), and psychosocial factors (i.e. stigma, anxiety, and depression) would be associated with general patient satisfaction. Hierarchical linear regression models were estimated to estimate general satisfaction scores from the Patient Satisfaction Short Form. Step 1 of the model controlled for demographic factors. Step 2 examined the role of disease characteristics above and beyond demographics. Finally, psychosocial factors were added in Step 3 to examine whether they were associated with patient satisfaction while controlling for demographic and disease characteristics.

Similarly, hierarchical linear regression models were estimated to examine the contribution of demographics, disease characteristics, and social factors to depression and anxiety symptoms in adults. In Step 1 of each model, the following demographic variables were entered: gender, race/ethnicity, income, and age. In Step 2 of each model, disease characteristics were added, including number of RDs, amount of time since diagnosis, disease course, PROMIS sleep disturbance, PROMIS physical function, PROMIS pain interference, PROMIS fatigue, and PROMIS ability to participate in social roles and activities. In Step 3 of each model, stigma was added in order to examine its effects when controlling for demographic and disease characteristics. Similar regression models were estimated for pediatric depression and anxiety, with a few exceptions: Step 1 did not include household income, as we expected this to be less relevant in the pediatric sample. The relevant PROMIS pediatric proxy scales were used for disease characteristics in Step 2, including PROMIS mobility, PROMIS pain, and PROMIS fatigue. Finally, PROMIS peer relationships was used instead of stigma in Step 3 because the latter has not been validated for pediatric proxy reporting.

## Results

### Sample characteristics

The final sample was predominantly adults living with RD (*n* = 787; 70%), female (*n* = 897; 80%), and white (*n* = 1007; 90%). The average age of individuals with RD in the sample was 40.90 years old (*SD* = 21.82 years; range = 1–90 years). Over 50% of people with RDs reported having at least a Bachelor’s degree and 25% of the sample reported earning an annual household income over $100,000 (Table 1). A total of 344 RDs were represented. The following disorders were the most frequently represented (Orphanet linearization classification is in parentheses): spinocerebellar ataxia (neurologic) (n = 178; 16%), idiopathic hypersomnia (neurologic) (n = 146; 13%), narcolepsy (neurologic) (n = 56; 5%), Ehlers-Danlos syndrome (systemic and rheumatologic) (n = 45; 4%), primary biliary cholangitis (hepatic) (n = 36; 3%), Mollaret meningitis (infectious) (n = 26; 2%), hypophosphatasia (rare developmental anomaly) (n = 23; 2%).

Approximately 70% of participants identified as an adult living with a RD (*n* = 787), 22% were a parent of a child with a RD (*n* = 252), and approximately 8% were relatives or caregivers to someone with a RD (*n* = 76). Participants reported that 80% of the individuals with the RD were adults (*n* = 893) and approximately 20% were children (under age 18; *n* = 234). Most participants reported only one RD (*n* = 995; 88%), though participants reported having up to 5 RD (9% reported 2 RDs, 2% reported 3 RDs, and 1% reported having 4 or 5 RDs; see Table 1 for descriptive statistics).

Nearly 80% of participants were female (*n* = 897), 19% were male (*n* = 219), and 1% identified as nonbinary or another identifier (*n* = 11). This is different from the gender composition of the people with RDs (about whom the participants were reporting), which was 66% female (*n* = 752), 32% male (*n* = 365), and 1% nonbinary or something else (*n* = 10). The sample of participants was mostly white (*n* = 1007; 90%), with participants of color representing the following race/ethnicity groups: Hispanic or Latinx (2%), Asian or Asian American (2%), Black or African American (2%), Mixed race (1%), Other (1%), and Middle Eastern, and Native Hawaiian or Pacific Islander (both less than 1%). Nearly half of adult participants with a RD (48%) were married or partnered, 33% were single, 10% were divorced or separated, 7% were dating or in a relationship, and 2% were widowed.

### Rare disease characteristics and diagnosis process

Most participants (60%) said that they had their RD symptoms for 10 years or more. Participants were divided across the different categories of disease progression: 34% indicated that their RD was stable, 44% said their RD was progressive, 22% said their RD was episodic, and 1% reported that their condition is expected to get better over time.

**Fig. 1:**
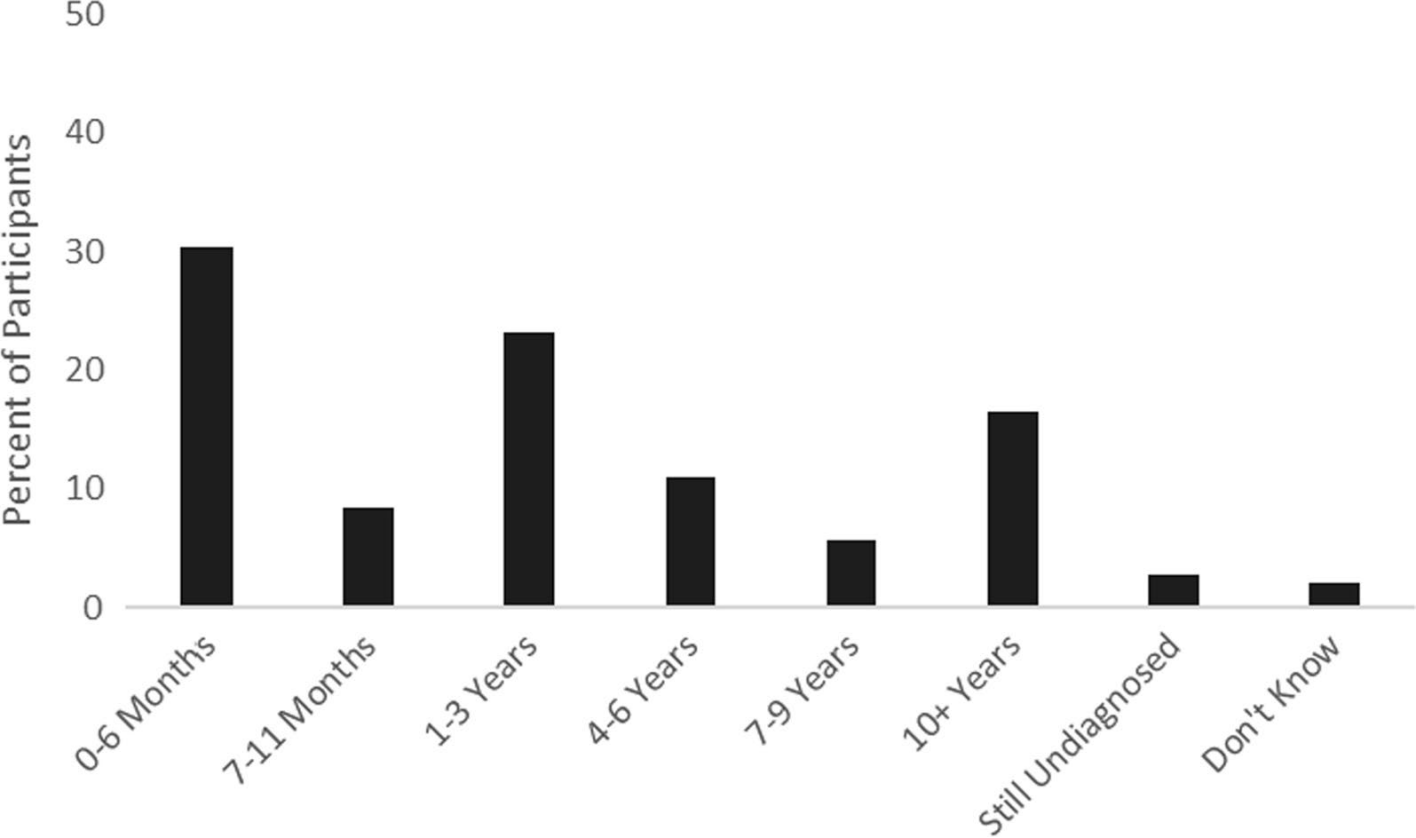
Self-reported time to get a confirmed diagnosis after seeking medical help

In addition to asking basic information about participants’ RD, the survey included questions designed to learn more about the process of receiving a diagnosis. Approximately 95% of this sample had a confirmed diagnosis, 4% had an unconfirmed diagnosis, and 1% had not been officially diagnosed with a RD. Participants with unconfirmed or unofficial diagnoses were included in this study if they self-reported a RD because previous research suggests that diagnostic delays are common for individuals with RDs [5, 11]. Indeed, participants reported widely varying times between the onset of their symptoms and their eventual diagnosis: 30% waited 0–6 months between symptoms and a diagnosis, 9% waited 7–11 months, 23% waited 1–3 years, 11% waited 4–6 years, 6% waited 7–9 years, and 16% waited 10 more years for a diagnosis (Fig. 1). Additionally, 3% of participants said that they are still waiting for a confirmed diagnosis and 2% did not know how long it took for them to be diagnosed. The majority of participants (91%) have had their RD for at least one year. There was also considerable variability in how many providers were seen during the diagnosis process: 16% of participants saw only 1 provider, 38% saw 2–3 providers, 24% saw 4–5 providers, 13% saw 6–10 providers, 3% saw 11–15 providers, and 5% saw more than 15 providers before being diagnosed. Specialists were most likely to have diagnosed this sample’s RD (19% of participants were diagnosed by a local specialist, 23% by a regional specialist, and 27% by a national specialist), followed by general practitioners (10%).

**Fig. 2:**
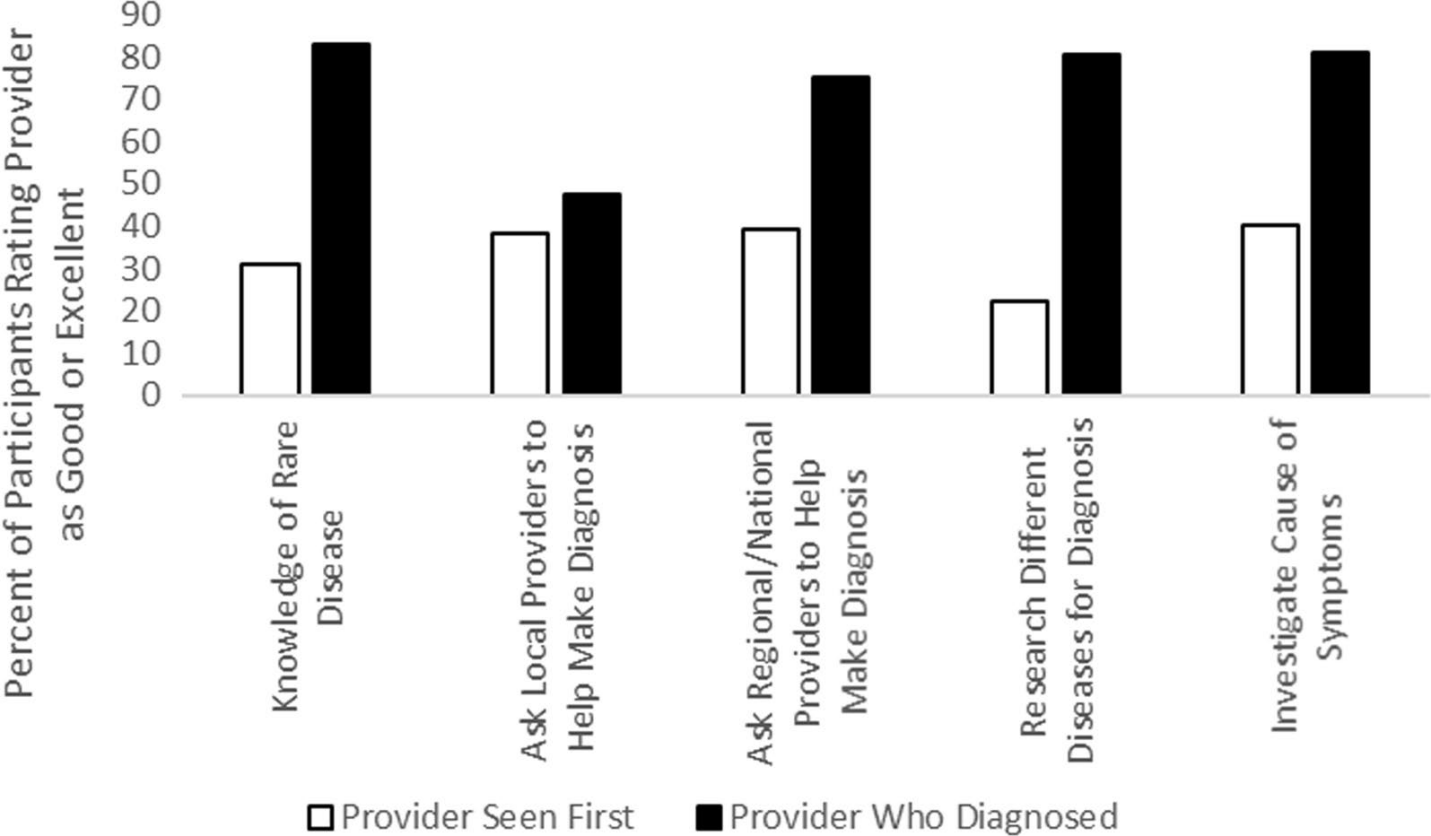
Participants’ ratings of providers’ knowledge of rare disease and willingness to seek support during diagnosis

The survey included questions asking participants to rate the provider they first saw about their symptoms and the provider who diagnosed them on characteristics related to the diagnostic process (Fig. 2). Nearly half of participants rated their initial provider as “poor” on their knowledge of RDs (45%), willingness to ask local physicians for help to make the diagnosis (46%), willingness to ask regional physicians for help to make the diagnosis (46%), and willingness to research different diseases to make a diagnosis (47%). Approximately 37% of participants also rated their initial provider as “poor” regarding their willingness to investigate causes of their symptoms, although 26% of participants rated their initial provider as “excellent” on this item. Perceptions of the providers who ultimately made the RD diagnosis were more positive: over half of participants rated their diagnosing provider as “excellent” on their knowledge of RDs (58%), willingness to ask local (54%) and regional/national physicians for help to make the diagnosis (54%), willingness to research different diseases (61%), and willingness to investigate the cause of their symptoms (65%). Thinking back to their diagnosis, about half of participants felt that they received enough information about their condition at diagnosis (51%) and most felt that they understood the information that they were given (64%).

### Health insurance

Most participants had at least one type of health insurance coverage, with the most common type being private health insurance (47% of the sample). The sample also included participants with Medicare (23%), Medicare supplements (10%), Medicaid (13%), Medicaid waivers (5%), military-related health care (e.g., VA, TriCare, 5%), and coverage from an ACA healthcare marketplace plan (3%). Approximately 17% of participants had dental insurance and 5% reported some other type of health insurance. Only 1% of the sample reported that they did not have any health insurance.

**Fig. 3:**
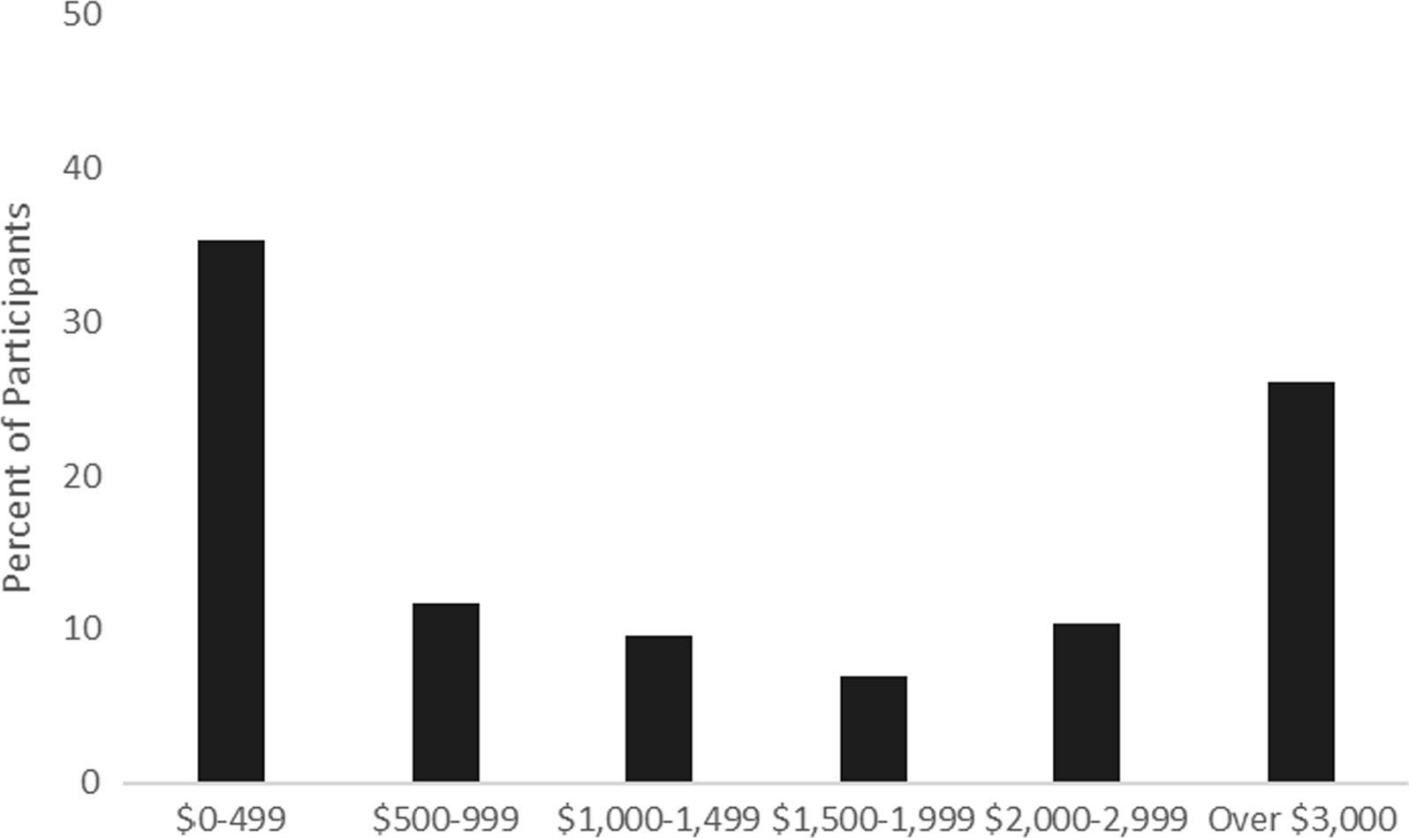
Self-reported out-of-pocket expenses for health claims in 2019 (USD)

Out-of-pocket health care expenses varied widely across participants: 35% reported paying $0–499 last year, 12% reported paying $500–999, 10% reported $1000–1499, 7% reported $1500–1999, 10% reported $2000–2999, and 26% reported paying over $3000 in out-of-pocket healthcare expenses last year (Fig. 3).

**Fig. 4:**
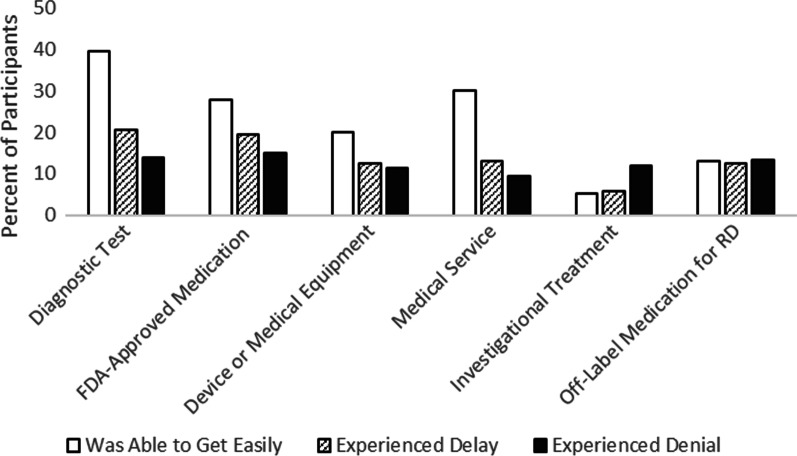
Experiences with US health insurance approvals, delays, and denials for RD tests, medications, treatments, and services

Participants also answered questions about if they have experienced delays or denial of treatment/care due to insurance issues (Fig. 4). Many participants reported easily getting diagnostic tests (40%), FDA-approved medications for treatment of their RD (28%), medical services (30%), and medical equipment or devices (20%) through their health insurance provider; fewer than 20% of participants experienced insurance-related delays or denials for any of these items. Fewer participants reported using investigational treatments (e.g., medications or treatments that are not FDA-approved or commercially available) or medications used for other reasons but not their particular condition. Among those, approximately 5% were able to get investigational treatments covered easily, 6% experienced delays, and 12% experienced denials; 13% were able to get off-label treatments easily, 13% experienced delays, and 13% experienced denials. The survey included additional questions asking about insurance denials for certain types of appointments and care. Approximately 14% of participants reported experiencing insurance denials to see specialists generally and 21% reported denials for seeing ‘out of network’ specialists. Approximately 11% of participants reported insurance denials for medical or dental procedures because there is no treatment for their RD and 14% reported denials of medical or dental procedures because there is not a defined ‘standard of care’ for their RD.

### Knowledge, care, and support

**Fig. 5:**
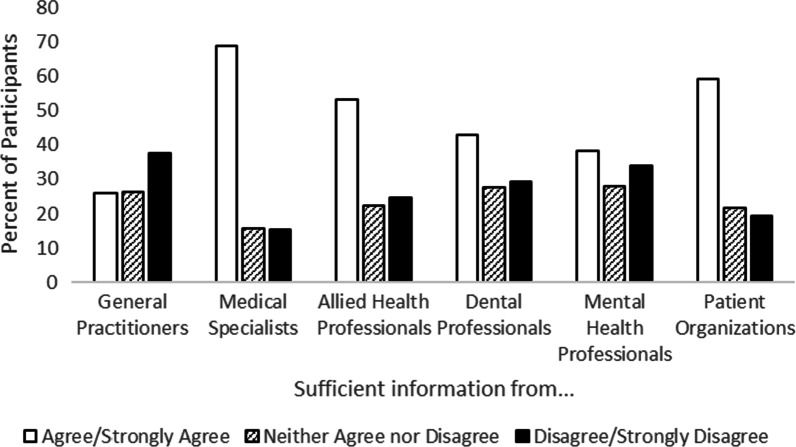
Perceptions of how well different provider types offered sufficient information about participants’ condition(s)

**Fig. 6:**
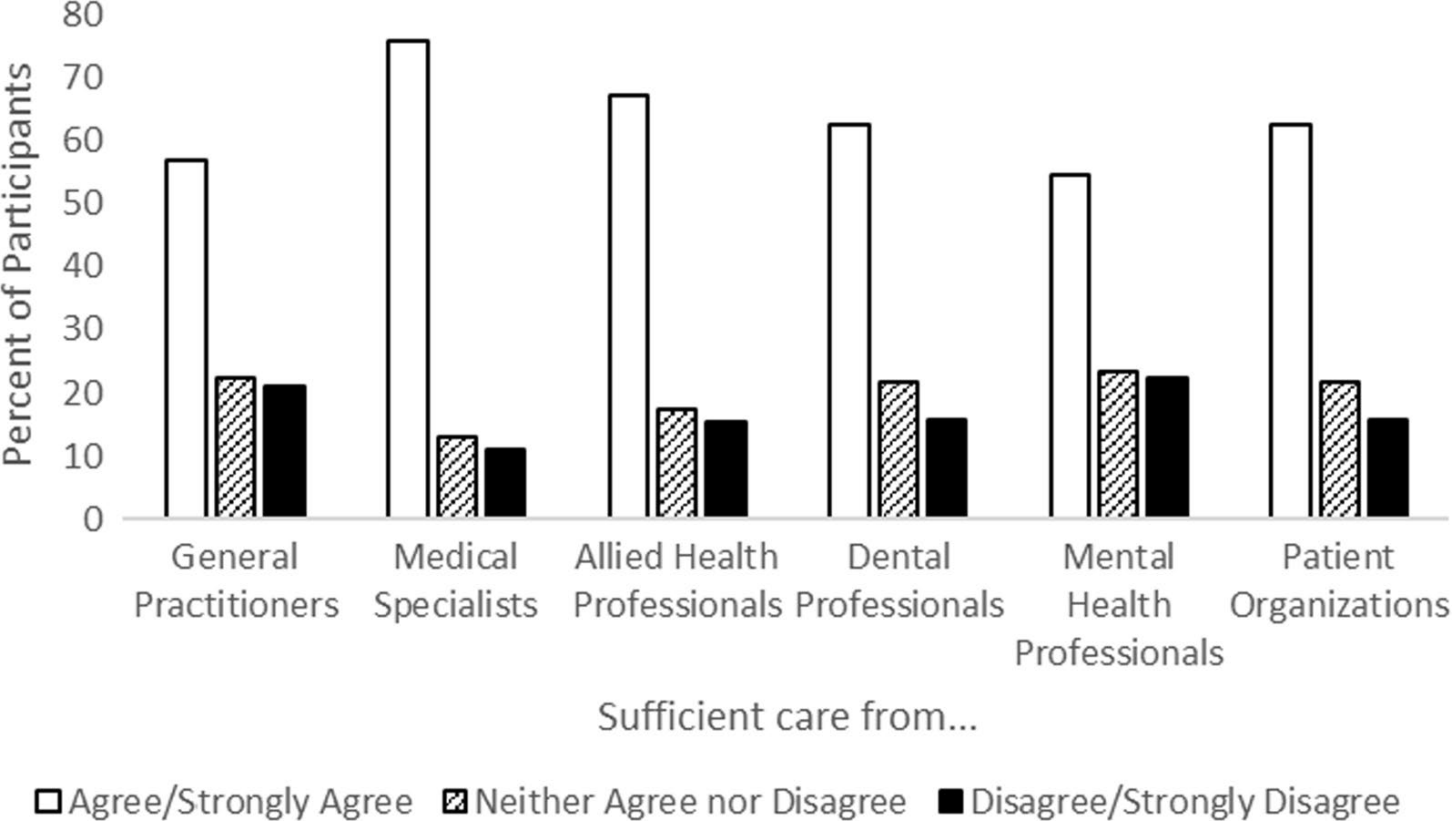
Perceptions of how well different provider types offered sufficient care for participants’ condition(s)

Participants were also asked to consider if the information and care that they received from different providers was sufficient. Many participants agreed or strongly agreed that the information they received from different providers was sufficient, although perceptions varied by provider type (Fig. 5): general practitioners (34%), specialists (69%), allied health professionals (53%), dentists (43%), and mental health professionals (39%), and from patient support organizations (59%). Perceptions of care were more positive than perceptions of information quality (Fig. 6). Many participants agreed or strongly agreed that the care they received from different providers was sufficient, including from general practitioners (57%), specialists (76%), allied health professionals (68%), dentists (63%), mental health professionals (55%), and from patient support organizations (63%).

**Fig. 7:**
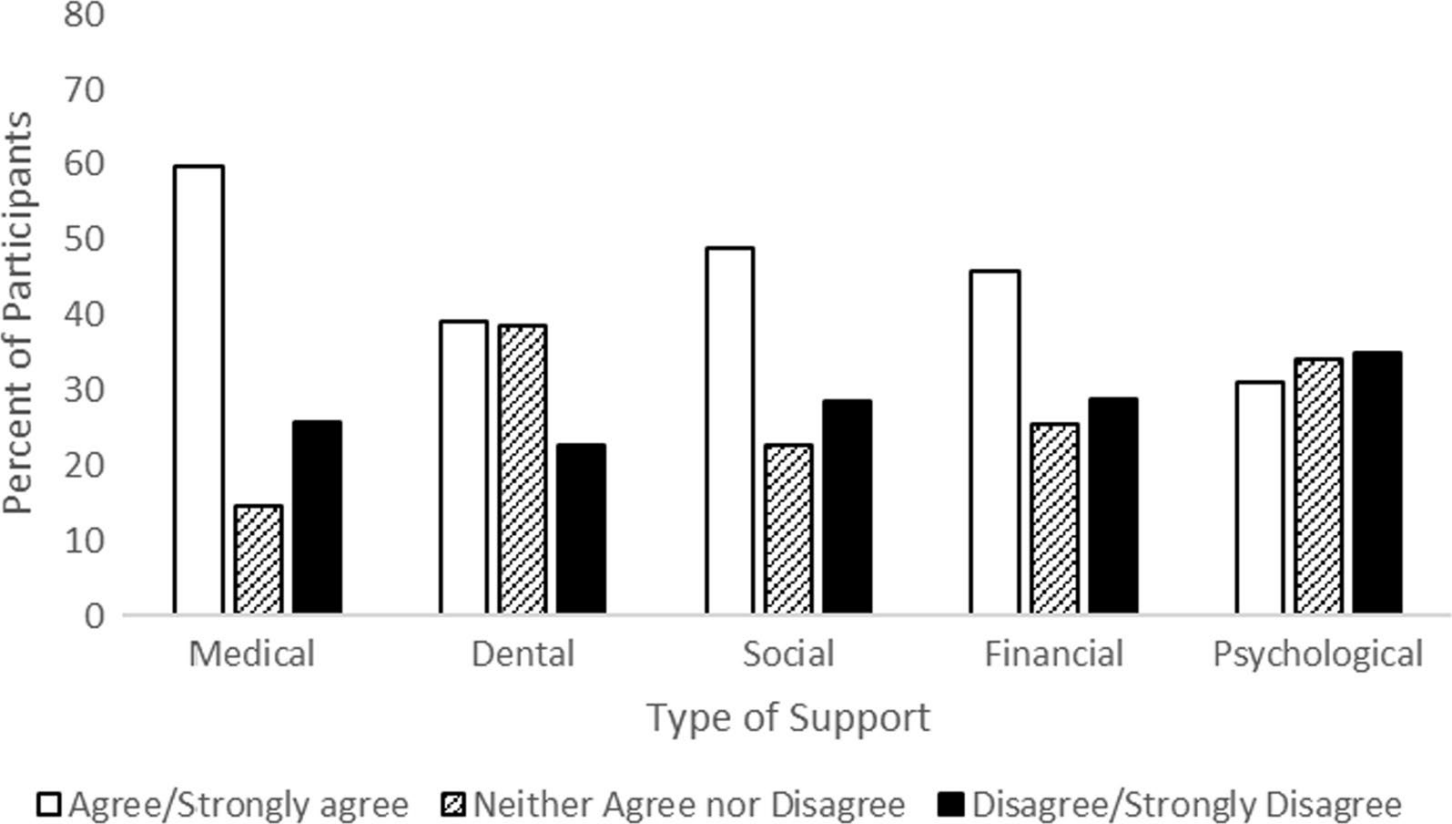
Participants’ perceptions of receiving sufficient types of support at diagnosis

Participants also considered their current knowledge about their RD and if the medical, dental, social, financial, psychological, and other support provided at the time of diagnosis was sufficient. On average, participants rated their knowledge as 7.5 out of 10 (*SD* = 1.89). Regarding the different types of support assessed in this study, 59% of participants agreed or strongly agreed that they received sufficient medical support, 39% agreed/strongly agreed that they received sufficient dental support, 49% agreed/strongly agreed that they received sufficient social support, 45% agreed/strongly agreed that they received sufficient financial support, 31% agreed/strongly agreed that they received sufficient psychological support, and 33% agreed/strongly agreed that the other support they received was sufficient (Fig. 7).

About 47% of participants were aware of a specialist center for their RD, but only 37% of participants said that they accessed a specialist center for their care. The most commonly-endorsed reasons for not using a specialist center for care is that there is not one (44%), it is too far away (18%), or something else (32%).

Even though specialist centers are not that common, the majority of participants (54%) reported that they have 1–2 different medical specialists to help them manage their RD. Approximately 24% of participants see 3–4 specialists, 9% see 5–6 specialists, and 12% see more than 6 specialists.

Nearly half of participants (47%) reported traveling over 60 miles for their RD care. Given the long distances that people may need to travel for care, it is not surprising that 58% of participants reported using telehealth services for appointments with specialists and 72% of participants expressed interest in using telehealth.

The use of care coordinators was not as common as the use of specialists and telehealth for managing RD. Only 12% of participants in the sample said that they have used a care coordinator. Among those who have used a care coordinator, the experience has been rated as helpful: 83% of participants rated them as a 7 or higher on a 1–10 scale of helpfulness, with 1 meaning not at all helpful and 10 meaning extremely helpful.

### Barriers to care and relocation

**Fig. 8:**
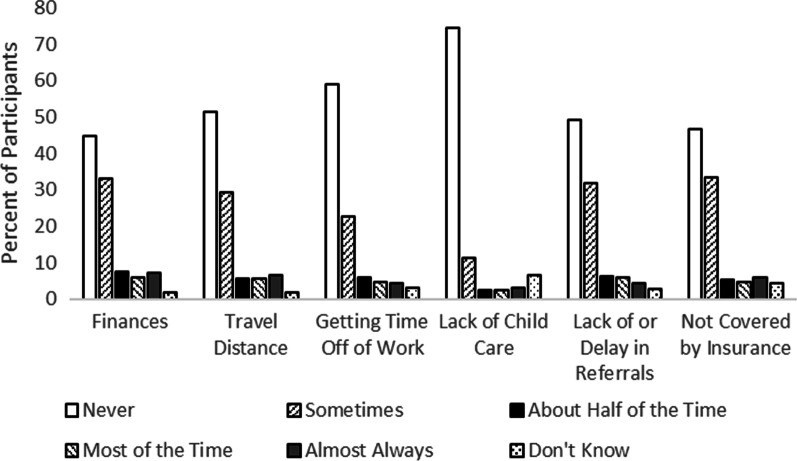
Participants’ experiences of different barriers to healthcare access

The survey also asked about barriers to care and if participants relocated to receive care for their RD. Participants were asked to consider if any of the following limited their ability to get medical or dental care for their RD (Fig. 8): finances, travel distance, difficulty getting time off of work, lack of childcare, lack of/delay in referrals, something not being covered by insurance, or something else. Regarding finances, the most common responses were that it never (45%) or sometimes (33%) affected their ability to get care. These were also the most common responses to the items asking about travel distance (never: 52%, sometimes: 29%), difficulty getting time off of work (never: 59%, sometimes: 23%), lack of childcare (never: 75%, sometimes: 11%), lack of/delay in getting referrals (never: 49%, sometimes: 32%), and for something not being covered by insurance (never: 47%, sometimes: 33%).

Very few participants reported that they relocated either within their state (3%) or outside their state (4%) to access treatment or clinical trials for their RD. Among these participants, it was more common to relocate permanently than temporarily.

### Provider types and satisfaction

Participants were asked if they saw different types of providers and then to rate their satisfaction with those providers. Patient satisfaction was assessed with both individual items asking about satisfaction and with a validated measure of satisfaction. Most participants visited a general practitioner (73%) and specialist (81%) in the past year. Other types of providers were less likely to be seen within the past year: 42% of participants visited an allied health professional (e.g., physical, speech, or occupational therapist), 33% visited a mental health professional, 45% visited a dentist, and 29% visited another type of provider (e.g., chiropractor, masseuse). To condense results, participants who reported that they were satisfied or very satisfied with the care they received on the single-item measures were combined. Most participants were satisfied with their general practitioners (62%) and specialists (74%). Fewer participants were satisfied with their allied health professionals (45%), dentists (44%), mental health professionals (35%), and other providers (28%).

Participants also rated their overall experience with healthcare providers using the Patient Satisfaction Short Form [5]. Mean scores were generally neutral (i.e. neither agree nor disagree) for the general satisfaction scale (*M* = 3.09, *SD* = 1.19), technical quality (*M* = 3.39, *SD* = 1.04), financial (*M* = 3.25, *SD* = 1.18), time spent with healthcare provider (*M* = 3.42, *SD* = 1.12), communication (*M* = 3.47, *SD* = 1.06), and accessibility (*M* = 3.29, *SD* = 1.00), with participants slightly satisfied with provider interpersonal manner (*M* = 3.90, *SD* = 0.93).

Assumptions for the linear regression of patient satisfaction were met, and the model is shown in Table 2. The addition of each step in the model resulted in a significant R^2^ change (*p*’s < 0.001). The final model was significant, *F*(18,562) = 15.08, *p* < 0.001, and explained 33% of the variance in patient satisfaction. Lower stigma, lower anxiety, shorter diagnostic odyssey, greater physical function, less pain interference, and less sleep disturbance were significant predictors of higher satisfaction in the final model.

**Table 2:** Regression predicting patient satisfaction in adults with RDs

Step	Predictor	b	Std. error	Beta	Sig.
1	Nonbinary (dummy coded 1 = nonbinary)	− 0.77	0.53	− 0.06	0.15
	Gender (dummy coded 1 = female)	− 0.24	0.11	− 0.09	0.03
	Race/ethnicity (1 = white, 2 = person of color)	− 0.12	0.17	− 0.03	0.48
	Income	0.05	0.02	0.08	0.06
	Age	0.01	0.00	0.16	0.00
2	Nonbinary (dummy coded 1 = nonbinary)	− 0.28	0.48	− 0.02	0.56
	Gender (dummy coded 1 = female)	− 0.03	0.10	− 0.01	0.81
	Race/ethnicity (1 = white, 2 = person of color)	0.00	0.16	0.00	1.00
	Income	0.01	0.02	0.02	0.64
	Age	0.01	0.00	0.13	0.00
	RD number	− 0.09	0.10	− 0.03	0.36
	Diagnostic delay	− 0.09	0.02	− 0.14	0.00
	Progressive course (dummy coded 1 = progressive)	− 0.15	0.11	− 0.06	0.18
	Episodic course (dummy coded 1 = episodic)	− 0.12	0.12	− 0.04	0.33
	Improving course (dummy coded 1 = improving)	− 1.11	0.61	− 0.07	0.07
	Sleep disturbance	− 0.02	0.01	− 0.15	0.00
	Physical function	− 0.01	0.01	− 0.11	0.03
	Pain interference	− 0.01	0.00	− 0.14	0.00
	Ability to participate in social roles and activities t score adults	− 0.02	0.01	− 0.17	0.00
	Fatigue	− 0.01	0.01	− 0.13	0.01
3	Nonbinary (dummy coded 1 = nonbinary)	− 0.12	0.46	− 0.01	0.80
	Gender (dummy coded 1 = female)	− 0.04	0.10	− 0.01	0.70
	Race/ethnicity (1 = white, 2 = person of color)	− 0.04	0.15	− 0.01	0.80
	Income	− 0.01	0.02	− 0.02	0.58
	Age	0.00	0.00	0.06	0.14
	RD number	− 0.06	0.09	− 0.02	0.54
	Diagnostic delay	− 0.08	0.02	− 0.12	0.00
	Progressive course (dummy coded 1 = progressive)	− 0.10	0.11	− 0.04	0.35
	Episodic course (dummy coded 1 = episodic)	− 0.04	0.12	− 0.01	0.74
	Improving course (dummy coded 1 = improving)	− 1.12	0.58	− 0.07	0.05
	Sleep disturbance	− 0.02	0.01	− 0.11	0.01
	Physical function	− 0.02	0.01	− 0.13	0.01
	Pain interference	− 0.01	0.00	− 0.11	0.01
	Ability to participate in social roles and activities t score adults	− 0.01	0.01	− 0.05	0.38
	Fatigue	− 0.01	0.01	− 0.07	0.18
	Stigma	− 0.03	0.01	− 0.21	0.00
	Anxiety	− 0.02	0.01	− 0.14	0.02
	Depression	− 0.01	0.01	− 0.07	0.24

### PROMIS, stigma, and comparisons to norms

PROMIS and stigma scores and comparisons to population norms can be viewed in Table 3. Adults with RDs had significantly poorer health-related quality of life and stigma in all domains compared to U.S. norms. Compared to adults with common chronic diseases, participants with RDs had poorer health-related quality of life in all domains in which norms were available; fatigue, *t*(694) = 14.44, *p* =  < 0.001; pain interference, *t*(698) = 2.45, *p* = 0.02; anxiety, *t*(694) = 7.94, *p* < 0.001, depression *t*(689) = 3.34, *p* < 0.001, physical function *t*(703) = -14.27, *p* < 0.001. Additional file 2 shows PROMIS and stigma scores for the two most frequent RDs in the sample. Similarly, children with RDs had poorer health-related quality of life compared to U.S. norms in all measured domains. Comparison norms of prevalent chronic conditions are not available in children.

**Table 3:** PROMIS and stigma scores among adults and children with RDs compared to population norms

Sample	Scale	M	SD
Adult			
	Ability to participate in social roles and activities	47.23**	9.62
	Anxiety	55.03**	10.18
	Depression	53.25**	10.10
	Fatigue	59.21**	12.19
	Pain interference	54.78**	11.27
	Physical function	40.16**	9.94
	Sleep disturbance	52.91**	8.77
	Stigma	55.47**	7.53
Pediatric proxy			
	Anxiety	53.47**	12.09
	Depression	53.90**	10.90
	Fatigue	56.06**	12.75
	Mobility	33.70**	10.77
	Pain interference	53.60**	11.69
	Peer relationships	44.38**	11.87

**p < .001

#### Regressions

Data met most regression assumptions, except a plot of standardized predicted values and standardized residuals suggested mild heteroscedasticity in both the anxiety and depression models. Thus, bootstrapping was used which is robust to violations of this assumption. Full models are shown in Table 4.

**Table 4:** Regressions predicting anxiety and depression in adults with RDs

Step	Predictor	Anxiety					Depression				
		b	SE	Sig.	Bootstrapped 95% confidence interval		b	SE	Sig.	Bootstrapped 95% confidence interval	
					Lower	Upper				Lower	Upper
1	Nonbinary (dummy coded 1 = nonbinary)	8.40	3.37	0.00	1.95	15.46	5.59	4.34	0.14	− 1.03	15.72
	Gender (dummy coded 1 = female)	2.00	0.99	0.05	− 0.01	4.17	0.97	0.91	0.29	− 0.73	2.93
	Race/ethnicity (1 = white, 2 = person of color)	0.18	1.50	0.90	− 2.94	2.93	− 1.30	1.49	0.37	− 4.14	1.58
	Income	− 0.65	0.21	0.00	− 1.06	− 0.25	− 1.10	0.21	0.00	− 1.50	− 0.67
	Age	− 0.13	0.03	0.00	− 0.18	− 0.08	− 0.08	0.03	0.00	− 0.13	− 0.03
2	Nonbinary (dummy coded 1 = nonbinary)	5.48	3.43	0.07	− 1.50	12.47	2.25	3.71	0.54	− 3.60	10.56
	Gender (dummy coded 1 = female)	− 0.26	0.87	0.76	− 1.93	1.53	− 0.97	0.81	0.23	− 2.46	0.65
	Race/ethnicity (1 = white, 2 = person of color)	− 0.79	1.33	0.55	− 3.46	1.79	− 2.73	1.22	0.02	− 5.17	− 0.23
	Income	− 0.33	0.19	0.07	− 0.68	0.05	− 0.71	0.18	0.00	− 1.05	− 0.34
	Age	− 0.10	0.02	0.00	− 0.15	− 0.05	− 0.05	0.02	0.00	− 0.10	− 0.01
	RD number	− 0.18	0.86	0.83	− 1.72	1.64	− 0.34	0.78	0.65	− 1.82	1.23
	Time since diagnosis	− 0.06	0.04	0.10	− 0.14	0.01	− 0.01	0.04	0.84	− 0.08	0.07
	Progressive course (dummy coded 1 = progressive)	1.88	0.90	0.04	− 0.04	3.64	0.49	0.88	0.55	− 1.18	2.35
	Episodic course (dummy coded 1 = episodic)	2.84	1.07	0.01	0.87	5.03	0.55	1.00	0.61	− 1.47	2.56
	Improving course (dummy coded 1 = improving)	3.69	3.03	0.17	− 1.53	10.07	3.70	4.15	0.36	− 2.60	12.73
	Sleep disturbance	0.18	.046^c^	0.00	0.09	0.27	0.16	0.05	0.00	0.07	0.26
	Physical function	0.09	0.05	0.07	0.00	0.18	0.00	0.05	0.93	− 0.09	0.08
	Pain interference	0.07	0.04	0.08	− 0.01	0.15	0.02	0.04	0.59	− 0.06	0.10
	Ability to participate in social roles	0.22	0.06	0.00	0.11	0.33	0.29	0.05	0.00	0.19	0.40
	Fatigue	0.18	0.04	0.00	0.10	0.26	0.20	0.04	0.00	0.13	0.28
3	Nonbinary (dummy coded 1 = nonbinary)	4.89	3.23	0.08	− 1.49	11.62	1.66	3.75	0.66	− 4.32	9.96
	Gender (dummy coded 1 = female)	− 0.24	0.86	0.78	− 1.87	1.51	− 0.95	0.79	0.23	− 2.38	0.64
	Race/ethnicity (1 = white, 2 = person of color)	− 0.76	1.33	0.56	− 3.44	1.81	− 2.70	1.20	0.02	− 5.00	− 0.25
	Income	− 0.23	0.18	0.21	− 0.59	0.12	− 0.61	0.18	0.00	− 0.96	− 0.25
	Age	− 0.08	0.02	0.00	− 0.13	− 0.03	− 0.03	0.02	0.12	− 0.08	0.01
	RD number	− 0.46	0.84	0.61	− 2.09	1.34	− 0.62	0.77	0.42	− 2.11	0.94
	Time since diagnosis	− 0.07	0.03	0.03	− 0.14	− 0.01	− 0.02	0.03	0.59	− 0.08	0.05
	Progressive course (dummy coded 1 = progressive)	1.79	0.88	0.05	− 0.10	3.50	0.40	0.87	0.62	− 1.29	2.28
	Episodic course (dummy coded 1 = episodic)	2.57	1.04	0.02	0.58	4.72	0.28	0.99	0.79	− 1.72	2.18
	Improving course (dummy coded 1 = improving)	4.51	1.87	0.01	0.81	8.29	4.49	2.13	0.02	0.45	8.85
	Sleep disturbance	0.17	0.05	0.00	0.08	0.26	0.15	0.05	0.00	0.06	0.24
	Physical function	0.12	0.05	0.02	0.02	0.21	0.02	0.05	0.63	− 0.07	0.11
	Pain interference	0.06	0.04	0.13	− 0.02	0.14	0.01	0.04	0.78	− 0.07	0.09
	Ability to participate in social roles	0.15	0.06	0.01	0.04	0.26	0.22	0.05	0.00	0.11	0.32
	Fatigue	0.17	0.04	0.00	0.09	0.25	0.19	0.04	0.00	0.12	0.26
	Stigma	0.26	0.06	0.00	0.15	0.38	0.26	0.06	0.00	0.14	0.37

Standard errors and confidence intervals based on 1000 bootstrap samples

##### Adults

###### Anxiety

The addition of each step resulted in a significant R^2^ change (p’s < 0.001). The final model was significant, *F*(16,577) = 21.15, *p* < 0.001. R^2^ indicated that the final model accounted for 37% of the variance in anxiety. Predictors of higher anxiety in the final model were younger age, less time since diagnosis, not having a stable disease course, more sleep disturbance, lower physical function, lower ability to participate in social roles and activities, more fatigue, and higher stigma.

###### Depression

The addition of each step resulted in a significant R^2^ change (p’s < 0.001). The final model was significant, *F*(16,576) = 22.02, *p* < 0.001. R^2^ indicated that the final model accounted for 38% of the variance in depression. Predictors of higher depression in the final model were being white, lower income, having an improving disease course, more sleep disturbance, lower ability to participate in social roles and activities, more fatigue, and higher stigma.

##### Children

Assumptions for linear regression were met, and the model is shown in Table 5 for regressions predicting anxiety and depression in children with RDs. The addition of each step after the first resulted in a significant R2 change (*p*’s < 0.01). The final models were significant for both anxiety, *F*(11,67) = 4.87, *p* < 0.001, explaining 49% of variance, and depression, *F*(11,58) = 7.36, *p* < 0.001, explaining 58% of variance. The same predictors of higher anxiety and depression emerged in the final models: more fatigue, poor peer relationships, and having fewer RDs.

**Table 5:** Regression predicting anxiety and depression in children with RDs

Step		Anxiety				Depression			
		b	Std. error	Beta	Sig.	b	Std. error	Beta	Sig.
1	Gender (dummy coded 1 = female)	− 0.96	3.25	− 0.04	0.77	− 3.38	2.84	− 0.15	0.24
	Race/ethnicity (1 = white, 2 = person of color)	0.28	4.50	0.01	0.95	− 0.97	3.78	− 0.03	0.80
	Age	0.69	0.43	0.21	0.11	0.49	0.38	0.17	0.19
2	Gender (dummy coded 1 = female)	− 1.23	2.91	− 0.05	0.67	− 1.64	2.36	− 0.07	0.49
	Race/ethnicity (1 = white, 2 = person of color)	0.94	3.75	0.03	0.80	− 0.73	2.91	− 0.02	0.80
	Age	0.22	0.44	0.06	0.63	0.23	0.35	0.08	0.52
	RD number	− 7.24	1.93	− 0.42	0.00	− 4.88	1.54	− 0.32	0.00
	Time since diagnosis	0.57	0.34	0.20	0.10	0.29	0.28	0.11	0.31
	Progressive course (dummy coded 1 = progressive)	− 1.44	3.62	− 0.05	0.69	− 4.59	2.83	− 0.19	0.11
	Episodic course (dummy coded 1 = episodic)	− 3.08	3.17	− 0.11	0.34	− 6.09	2.56	− 0.25	0.02
	Mobility	− 0.01	0.16	− 0.01	0.95	0.16	0.13	0.15	0.21
	Pain interference	0.23	0.16	0.20	0.15	0.07	0.12	0.07	0.55
	Fatigue	0.47	0.14	0.48	0.00	0.52	0.11	0.60	0.00
3	Gender (dummy coded 1 = female)	0.43	2.89	0.02	0.88	− 0.13	2.31	− 0.01	0.96
	Race/ethnicity (1 = white, 2 = person of color)	1.55	3.61	0.04	0.67	− 0.16	2.77	− 0.01	0.95
	Age	0.19	0.42	0.06	0.66	0.21	0.34	0.07	0.54
	RD number	− 6.87	1.87	− 0.40	0.00	− 4.53	1.47	− 0.29	0.00
	Time since diagnosis	0.55	0.33	0.19	0.11	0.26	0.26	0.10	0.33
	Progressive course (dummy coded 1 = progressive)	0.47	3.57	0.02	0.89	− 2.85	2.77	− 0.12	0.31
	Episodic course (dummy coded 1 = episodic)	− 1.62	3.11	− 0.06	0.60	− 4.76	2.48	− 0.19	0.06
	Mobility	− 0.02	0.16	− 0.02	0.89	0.15	0.12	0.14	0.22
	Pain interference	0.16	0.15	0.14	0.31	0.01	0.12	0.01	0.94
	Fatigue	0.32	0.15	0.32	0.03	0.38	0.12	0.44	0.00
	Peer relationships	− 0.33	0.14	− 0.31	0.02	− 0.30	0.11	− 0.31	0.01

## Discussion

This large-scale survey explored demographics, many aspects of healthcare access, patient satisfaction, and health-related quality of life among people with RDs in the U.S. The sample represented a total of 344 different RDs. In line with previous research, some participants experienced significant diagnostic delay [5, 11]. About one third waited four or more years for a diagnosis, and nearly a quarter saw six or more doctors in order to receive a diagnosis, similar to Molster and colleagues’ findings in Australian adults [5]. Approximately half of participants also reported misdiagnosis during their diagnostic odyssey, which replicates findings from a survey of people with RD in the United Kingdom [2]. The majority of participants in the current sample had at least one type of health insurance; even with insurance, approximately one quarter of this sample paid $3000 or more out-of-pocket for healthcare expenses. Participants’ experience with their health insurance varied, with some participants reporting insurance-related delays or denials for tests, treatments, services, and specialist appointments.

Participants primarily visited general practitioners and specialists for care, though it was relatively common for participants to visit other types of healthcare providers as well. Similar to Molster and colleagues’ findings, even though the majority of participants reported that they did not access a specialist center for their RD, most participants reported having one or two specialists who provide care for their RD [5]. Nearly half of participants in this study reported traveling at least 60 miles for RD care, while only a quarter of Australian adults with RDs traveled this far [5]. Also indicative of the challenges of having RDs, many participants reported seeing three or more specialists for RD care, but only a small minority of participants had a care coordinator, which is also consistent with findings from Molster et al. [5]. Many participants reported accessing care via telehealth or were interested in receiving some care through telehealth, which may help alleviate travel burdens. Perhaps one silver lining of the disruption of medical care due to the COVID-19 pandemic will be increased ubiquity of telehealth visits, which may be especially helpful for individuals with RDs who often face significant travel to receive appropriate healthcare.

Considering the information they received from different providers, participants were most likely to agree that information received from specialists was sufficient and were less likely to report that the information they received from their general or primary care doctor was sufficient, which is consistent with studies from Australia and Europe [5, 6]. Similar to Molster’s findings, information from patient support organizations was perceived more positively than information from some types of health providers [5]. This is not surprising given that support organizations offer information tailored to a specific condition and focus on patient experiences and perspectives [3].

Additionally, participants were often most satisfied with their specialists. Findings from the Patient Satisfaction Short Form suggested that participants were generally not satisfied with their other providers, who may have less knowledge and awareness of RDs than specialists [22]. Predictors of patient satisfaction were lower stigma, lower anxiety, shorter diagnostic odyssey, greater physical function, less pain interference, and less sleep disturbance. Thus, patients are more satisfied with their providers when they experience greater health-related quality of life and less stigma.

Around half of participants felt their medical and social support was sufficient, yet less than a third had sufficient dental and psychological support. Together, these findings indicate that a majority of people with RDs are not getting sufficient support. Findings from the current study are similar to what Limb et al. reported in their RD survey [2], which suggests that there is room for growth when considering how to support individuals with RDs and their families. Providers and patient support organizations could focus additional efforts on outreach regarding these types of support.

Regarding potential barriers to healthcare, a subset of participants identified finances, travel distance, difficulty getting time off work, lack of childcare, lack of/delay in getting referrals, and health insurance issues as potential barriers. While these may be barriers to care for individuals with any type of health condition, they may have a bigger impact on individuals with RD because they may already be facing barriers due to diagnostic issues and low satisfaction with providers.

Health-related quality of life findings replicated and extended Bogart and Irvin’s (2017) study [11], finding that not only adults, but also children, with RDs had significantly poorer health-related quality of life on all domains in which U.S. population and common chronic disease norms were available. Similarly, adults with RDs experienced greater stigma than those with prevalent chronic conditions. Importantly, this study extended previous findings by examining predictors of anxiety and depression. For adults, stigma, fatigue, lower ability to participate in social roles, sleep disturbance, and not having a stable disease course were associated with anxiety and depression. It is noteworthy that not having a stable disease course was associated with anxiety, pointing to the stressful experience of having an unpredictable condition [23]. For children, poor peer relationships, fatigue, and having fewer RDs were associated with anxiety and depression. The role of peer relationships in children’s mental health parallels the findings in adults that being able to participate in social roles and having lower stigma were associated with better mental health. On the other hand, the finding that having fewer RDs was associated with poorer mental health in children was unexpected. One interpretation could be that children with multiple RDs (or their parents) have different or lower expectations for health-related quality of life. However, the sample reporting children's mental health was small, so these findings should be considered tentative.

### Limitations and strengths

This survey was cross-sectional in nature, so causality cannot be inferred. Due to the dearth of epidemiological studies characterizing populations or prevalence of RD in general (i.e., across specific diagnoses), it is difficult to know how representative this sample is of the larger population of people diagnosed with RDs in the U.S. This study successfully recruited individuals with a variety of RDs, but our sample is likely to have had more white respondents, and was likely more educated and more affluent than the actual population of individuals with RDs in the U.S. Future surveys should be designed and circulated to recruit more diverse samples. Data collection for the study occurred during the COVID-19 pandemic. Participants were instructed to consider their experiences before COVID-19 when answering survey questions, but results are likely to be influenced by the pandemic anyway. While this limitation needs to be considered, results for many of the variables we measured were consistent with previous, pre-pandemic RD surveys. One notable discrepancy is that use of telehealth was much higher than has been reported in other research [5], which may be an artifact of the increased use of telehealth during the pandemic.

An additional limitation is that proxy reports (e.g., from parents, relatives, or caregivers) may not accurately represent the experiences of individuals with RDs. We used validated proxy reports when possible (e.g., PROMIS), but it is still possible that parent/caregiver impressions of healthcare and health-related quality of life differ from the experiences of individuals with RDs.

Despite these limitations, to our knowledge, this is one of the largest studies of healthcare access and health-related quality of life among people with RDs conducted in the U.S. This sample size provided the statistical power to use regression to predict anxiety, depression, and patient satisfaction to learn more about how RD characteristics, healthcare experiences, and psychosocial factors are related to health-related quality of life.

### Implications

This study suggests that there are many shared experiences among individuals with RDs despite their different diagnoses. Barriers and dissatisfaction with healthcare were commonly reported. Additionally, psychosocial concerns were commonly reported by participants. Healthcare providers and patient support organizations should focus on reducing stigma and providing additional knowledge and support for individuals with RDs and their families. One of the advantages of focusing on reducing stigma and improving social support for individuals with RDs in future work is that there is already a rich literature investigating these topics that can be used to inform work in the RD space [24, 25]. An additional advantage is that these types of interventions can be delivered regardless of which specific RD(s) participants are managing. Future research could develop or adapt stigma, social support, and mental health interventions for the RD community. These findings indicate that much advocacy work is still needed in order to improve healthcare access and ultimately health-related quality of life.

## Conclusions

Participants with RDs in the United States experienced significant diagnostic delays. The majority of participants had health insurance, yet more than one quarter paid $3000 or more out of pocket in the previous year. Although most participants did not access a specialist center, the majority see at least one or two specialists for their RD. Participants were dissatisfied with their healthcare providers in general, but were more satisfied with their specialists. About half of participants traveled more than 60 miles for care and/or accessed their specialists via telehealth. These healthcare challenges take a notable toll on the health-related quality of life of individuals with RDs, resulting in generally poorer health-related quality of life compared to average Americans and Americans with common chronic diseases. Fatigue and stigma (or peer relationships for children) were associated with depression and anxiety in this sample when controlling for demographics and other disease characteristics, suggesting that these psychosocial factors that cut across etiologies may be important for understanding the experiences of people with RDs. Future work can address these common areas of concern across RDs to improve health-related quality of life for both adults and children with RDs in the U.S.

## Supplementary Information

**Additional file 1:** Rare Disease Healthcare and Health-Related Quality of Life survey.

**Additional file 2:** PROMIS and stigma scores for the two most frequent RDs in the sample.

## Data Availability

The datasets used and/or analyzed during the current study are available from the corresponding author on reasonable request.

## Footnotes

1 This study was conducted as part of research by the Chloe Barnes Advisory Council on Rare Disease in the state of Minnesota. Findings did not appreciably differ between those residing in Minnesota and the rest of the United States, so results from the combined sample are reported here to maximize statistical power.
