# Supplementary material for: Healthcare access, satisfaction, and health-related quality of life among children and adults with rare diseases
Source: Orphanet J Rare Dis. 2022 May 12;17:196. doi: 10.1186/s13023-022-02343-4 (PMC9096775; doi:10.1186/s13023-022-02343-4)
Supplement: Supplementary file 1 — Additional file 1: Rare Disease Healthcare and Health-Related Quality of Life survey. [file 13023_2022_2343_MOESM1_ESM.docx]

Supplemental Material

Rare Disease Health Care Access Survey

*(Exported from Qualtrics; formatting may differ)*

Start of Block: Demographics

Thank you for agreeing to take our survey! We will ask you a series of questions about your/your family member's background, rare disease, the way you/they obtain care and information about your disease, and your/their socioemotional concerns. 


We are interested in understanding your experience accessing care BEFORE the COVID-19 pandemic. There will be a question near the end of the survey to ask about your experience related to COVID-19 where you can share how things may have changed or stayed the same. 


For the purpose of this survey: A caregiver is a person who (usually without being paid) provides ongoing care or assistance to another person living with a rare disease. Caregivers are often family members of the person living with a rare disease. A paid support worker is a person who is paid to provide ongoing care or assistance to another person living with a rare disease. 
In this survey, we use the phrases “the person with the rare disease” or "you/your family member" to refer to the person living with a rare disease. If you are a paid support worker, please interpret this as the person you provide care for.

Is the person answering this survey:

- A person living with a rare disease (1)
- A relative/caregiver of a person with a rare disease (2)
- A parent of a child with a rare disease (3)
- A paid support worker (4)
- Other (please describe) (5) ________________________________________________

Is the person answering this survey aged 18 years or older?

- Yes (1)
- No - This survey should be completed by an adult at least 18 years old (2)

Is the person answering the survey:

- Female (1)
- Male (2)
- Something else (please describe) (4) ________________________________________________

Has the person with the rare disease recovered from the disease or died?

- Yes - This survey should be completed by someone with current experience with rare disease (1)
- No (2)

Is the person with the rare disease a child (under 18) or an adult (18 or older)?

- Child (1)
- Adult (2)

What year was the person with the rare disease born?

▼

What is the race/ethnicity of the person with the rare disease? Select all that apply.

- American Indian or Alaska native (1)
- Asian (2)
- Black or African American (3)
- Hispanic or Latino/a (4)
- Middle Eastern (5)
- Mixed race (6)
- Native Hawaiian or Pacific Islander (7)
- White or Caucasian (8)
- Other (9) ________________________________________________
- Rather not say (10)
- Don't know (12)

Is the person with the rare disease:

- Female (1)
- Male (2)
- Something else (please describe) (4) ________________________________________________

What is the marital/partnership status of the person with the rare disease?

- Single (never married or partnered) (1)
- Dating/in a relationship (2)
- Married or partnered (3)
- Divorced or separated (4)
- Widowed (5)
- Don't know (6)
- Not applicable (7)

What is the highest level of education obtained by the person with the rare disease?

- Some high school or less (1)
- High school diploma or GED (2)
- Some college (3)
- Associates or technical degree (4)
- Bachelor's degree (5)
- Some graduate school (6)
- Graduate degree (7)
- Don't know (8)
- Not applicable (9)

What was the approximate household income (USD) in 2019, before taxes, of the person with the rare disease?

- Under $20,000 (1)
- $20,000-39,000 (2)
- $40,000-59,000 (3)
- $60,000-79,000 (4)
- $80,000-99,999 (5)
- Over $100,000 (6)
- Rather not say (7)
- Don't know (8)
- Not applicable (9)

The person with the rare disease lives in a:

- Large city (>500,000 residents) (1)
- Medium city (100,000-500,000 residents) (2)
- Small town or city ( (3)
- Rural area (outside of a town or city) (4)
- Don't know (5)

What state does the person with the rare disease live in?

▼

What county does the person with the rare disease live in?

▼

Does the person with the rare disease: 
(Select all that apply)

- Work full-time (1)
- Work part-time (2)
- Volunteer (3)
- Attend school/college/university (4)
- Not work because you/they are not able (5)
- Not work because you/they choose not to (6)
- Not work because you/they are unable to find job (7)
- Don't know (8)
- Not applicable (9)

End of Block: Demographics

Start of Block: RD questions

Next, we'll ask questions about your/your family member's rare disease(s).

 A disorder/disease is generally considered rare if it affects fewer than 200,000 individuals in the United States. A list of rare diseases can be found [here](https://rarediseases.info.nih.gov/diseases/browse-by-first-letter). Rare disorders are discovered and prevalence estimates change frequently, so even if your disorder does not appear in the list, you may write it in below.

How many rare diseases do you/your family member have?

- 1 (1)
- more than 1 (please write number here) (2) ________________________________________________
- Don't know (3)

What is the name of your/your family member's rare disease(s)?

________________________________________________________________

End of Block: RD questions

Start of Block: Health follow-up questions/diagnosis

Select the statement below that best describes the time course of your/your family member's rare disease(s).

- It is stable; not expected to change very much over time (1)
- It is progressive; expected to get more severe over time (2)
- It is episodic; I have periods of time when I am stable or get better followed by periods when I get worse. (3)
- It is improving; it is expected to get better over time. (4)
- Don't know (5)

How long have you/your family member experienced symptoms of the rare disease(s)?  If you have more than one rare disease, respond about the one you have had the longest.

- 0-6 months (1)
- 7-11 months (2)
- 1-3 years (3)
- 4-6 years (4)
- 7-9 years (5)
- 10+ years (6)
- Don't know (7)

How long after first seeking medical help did it take for you/your family member to get a confirmed diagnosis?  If you have more than one rare disease, respond about the one you have had the longest.

- 0-6 months (1)
- 7-11 months (2)
- 1-3 years (3)
- 4-6 years (4)
- 7-9 years (5)
- 10+ years (6)
- Still undiagnosed (7)
- Don't know (8)

Have you/your family member received a diagnosis of this condition confirmed by a doctor(s)?

- Yes, I/they have a confirmed diagnosis (1)
- No, I/they have an unconfirmed diagnosis (2)
- No, I/they do not have a diagnosis (3)
- Don’t know (4)

How many doctors have you/your family member seen to get a confirmed diagnosis? If you/your family member have not received a confirmed diagnosis, how many doctors have you seen so far in your attempt to get a diagnosis?

- 1 (1)
- 2-3 (8)
- 4-5 (2)
- 6-10 (3)
- 11-15 (4)
- More than 15 (5)
- Don't know (6)
- Not applicable (7)

How long ago was the confirmed diagnosis made?

- Less than a year ago. Please indicate how many months ago. (1) ________________________________________________
- More than a year ago. Please indicate how many years ago. (2) ________________________________________________
- Don't know (3)
- Not applicable (4)

Who diagnosed the person with the rare disease’s rare condition? Select all that apply.

- General practitioner/primary care doctor (1)
- Allied health professionals (e.g., optician, physical therapist, podiatrist, speech therapist, nutritionist, occupational therapist) (2)
- Local medical specialists in Minnesota (e.g., specialist doctors). Please describe (e.g., geneticist, neurologist, allergist) (3) ________________________________________________
- Regional medical specialists in the Midwest (e.g., specialist doctors). Please describe (e.g., geneticist, neurologist, allergist) (4) ________________________________________________
- National medical specialists outside the Midwest (e.g., specialist doctors). Please describe (e.g., geneticist, neurologist, allergist) (5) ________________________________________________
- Hospital emergency department (6)
- Hospital outpatient services/clinics (not specialist centers in your/your family member’s condition) (7)
- Dental services (e.g., dentist, dental hygienist) (8)
- Mental health service (e.g., psychiatrist, psychologist, counselor) (9)
- Alternative health service (e.g., acupuncturist, naturopath, homeopath, or any other alternative health service) (10)
- Other (please describe) (11) ________________________________________________
- Don't know (12)
- Not applicable (13)

Sometimes people with rare disease(s) experience situations related to the diagnosis, management or treatment of their rare disease that have felt difficult or frustrating.  These are often called “barriers”.  Reflecting on your own situation, please describe your MOST SIGNIFICANT barrier that you have experienced. Please be as specific as possible. (If there are none, please note “none”):

________________________________________________________________

Please describe your second most significant barrier:

________________________________________________________________

Please describe your third most significant barrier:

________________________________________________________________

Sometimes people with rare disease(s) experience situations related to the diagnosis, management or treatment of their rare disease that have been positive or helpful. These are often called "facilitators". Reflecting on your own situation, please describe the MOST HELPFUL support that you have experienced. Please be as specific as possible. (If there are none, please note “none”):

________________________________________________________________

Please describe your second most significant facilitator:

________________________________________________________________

Please describe your third most significant facilitator:

________________________________________________________________

Please rate the provider first seen at the onset of symptoms, and also the provider who made the diagnosis. If the provider seen first made the diagnosis, only answer the questions for Provider who made diagnosis. 

How would you rate their...

|  | Provider seen first | | | | | | Provider who made diagnosis | | | | | |
| --- | --- | --- | --- | --- | --- | --- | --- | --- | --- | --- | --- | --- |
|  | Poor (1) | Fair (2) | Neutral (3) | Good (4) | Excellent (5) | Don't know/Not applicable (6) | Poor (1) | Fair (2) | Neutral (3) | Good (4) | Excellent (5) | Don't know/Not applicable (6) |
| Knowledge of rare disease? (1) |  |  |  |  |  |  |  |  |  |  |  |  |
| Willingness to ask other local physicians (from Minnesota) to help make a diagnosis? (2) |  |  |  |  |  |  |  |  |  |  |  |  |
| Willingness to ask regional or national (in or outside the Midwest) experts to help make a diagnosis? (3) |  |  |  |  |  |  |  |  |  |  |  |  |
| Willingness to research different diseases to make a diagnosis? (4) |  |  |  |  |  |  |  |  |  |  |  |  |
| Willingness to investigate the cause of symptoms? (5) |  |  |  |  |  |  |  |  |  |  |  |  |

Looking back, at the time of confirmed diagnosis, do you think you/your family member were provided enough information on your/their condition?

- Yes (1)
- No (2)
- Don't know (3)

Did you/your family member understand all of the information you were given about your/their condition(s) at the time of diagnosis?

- Yes (1)
- No. What information didn't you understand? (Please describe) (2) ________________________________________________
- Don't know (3)

What other comments do you have about you/your family member being diagnosed with a rare condition? What information was helpful/unhelpful? Was there information that you wish you had received but didn’t?

________________________________________________________________

________________________________________________________________

________________________________________________________________

________________________________________________________________

________________________________________________________________

End of Block: Health follow-up questions/diagnosis

Start of Block: Insurance

Thank you. Next are some questions about health insurance.

What kind, if any, of health insurance or health care coverage does the person with the rare disease have? Select all that apply.

- Private insurance (1)
- Healthcare marketplace insurance (ACA or Obamacare) (2)
- Dental insurance (3)
- Medicare (for US residents) (4)
- Medicare supplement (for US residents) (5)
- Medicaid (for US residents) (6)
- Medicaid waiver (7)
- Military related health care including VA health care, TriCare, and CHAMP-VA (8)
- Other (please describe) (9) ________________________________________________
- The person with the rare disease does NOT have health care coverage (10)
- Don't know (11)

You/your family member paid approximately _____ (USD) out-of-pocket (of your/their own money) in the 2019 calendar year for health claims (physician visit co-pays, deductibles, co-insurance, prescriptions, TEFRA):

- $0-$499 (1)
- $500-$999 (2)
- $1,000-$1,499 (3)
- $1,500-$1,999 (4)
- $2,000-$2,999 (5)
- Over $3,000 (6)
- Don't know (7)

Have you/your family member ever been denied or delayed any of the following because pre-approval from health insurance was required?

|  | Was able to get easily (1) | Experienced delay (2) | Experienced denial (3) | Don't know/Not applicable (4) |
| --- | --- | --- | --- | --- |
| Diagnostic test (1) |  |  |  |  |
| Medication that is FDA approved or commercially available for your/your family member's condition (2) |  |  |  |  |
| Device or medical equipment (3) |  |  |  |  |
| Medical service (e.g., occupational therapy) (4) |  |  |  |  |
| Investigational treatment (e.g., medication that is NOT FDA approved or commercially available) (5) |  |  |  |  |
| Medication that is used for other reasons but not your/your family member's particular condition (e.g., off-label use or for different age groups) (6) |  |  |  |  |

Has your/your family member's insurance ever denied referral to a medical or dental specialist?

- Yes (please describe) (1) ________________________________________________
- No (2)
- Don't know (3)

Has your/your family member's insurer ever denied covering a medical or dental procedure or test due because there is no available treatment for the rare disease?

- Yes (please describe) (1) ________________________________________________
- No (2)
- Don't know (3)

Has your/your family member's insurer ever denied you a medical or dental service/procedure due to the rare disease not having a defined "standard of care"?

- Yes (please describe) (1) ________________________________________________
- No (2)
- Don't know (3)

Have you/your family member ever been denied seeing a specialist in your rare disease due to that specialist being "out of network"?

- Yes (please describe) (1) ________________________________________________
- No (2)
- Don't know (3)

End of Block: Insurance

Start of Block: Knowledge and support

Thank you. Next, we have some questions about the information, support, and care that the person with the rare disease received about their condition.

Have you/your family member had sufficient information and care for the rare disease from each of the following sources?

|  | Sufficient information? | | | | | | Sufficient care? | | | | | |
| --- | --- | --- | --- | --- | --- | --- | --- | --- | --- | --- | --- | --- |
|  | Strongly disagree (1) | Disagree (2) | Neutral (3) | Agree (4) | Strongly agree (5) | Don't know/Not applicable (6) | Strongly disagree (1) | Disagree (2) | Neutral (3) | Agree (4) | Strongly agree (5) | Don't know/Not applicable (6) |
| General practitioner (GP) (1) |  |  |  |  |  |  |  |  |  |  |  |  |
| Medical specialists (2) |  |  |  |  |  |  |  |  |  |  |  |  |
| Allied health professionals (e.g., physical/physio therapist, occupational therapist, speech therapist) (3) |  |  |  |  |  |  |  |  |  |  |  |  |
| Dental professional (4) |  |  |  |  |  |  |  |  |  |  |  |  |
| Mental health professional (e.g., social worker, psychologist, psychiatrist) (5) |  |  |  |  |  |  |  |  |  |  |  |  |
| Patient organizations (e.g., charities) (6) |  |  |  |  |  |  |  |  |  |  |  |  |

Do you think you now have sufficient knowledge about your/your family member’s rare condition(s)? Rate your knowledge from 1 (no knowledge) to 10 (complete knowledge):

- 1 (9)
- 2 (10)
- 3 (11)
- 4 (12)
- 5 (13)
- 6 (14)
- 7 (15)
- 8 (16)
- 9 (17)
- 10 (18)

If you rated your knowledge as less than 10, specifically, what information do you feel you are missing?

________________________________________________________________

Do you agree or disagree that you/your family member received sufficient support in the following areas at the time of diagnosis?

|  | Strongly disagree (1) | Disagree (2) | Neutral (neither agree nor disagree) (3) | Agree (4) | Strongly agree (5) | Don't know (6) | Not applicable (7) |
| --- | --- | --- | --- | --- | --- | --- | --- |
| Medical (for example, doctors, nurses) (1) |  |  |  |  |  |  |  |
| Dental (2) |  |  |  |  |  |  |  |
| Social (for example, family, friends, church members) (3) |  |  |  |  |  |  |  |
| Financial (for example, insurance coverage) (4) |  |  |  |  |  |  |  |
| Psychological (for example, mental health care, counselors) (5) |  |  |  |  |  |  |  |
| Other (please specify) (6) |  |  |  |  |  |  |  |

End of Block: Knowledge and support

Start of Block: Specialist centers/care

A 'specialist center' is a center or clinic that is able to provide expert advice on diagnosis, assessment and treatment of a particular condition or group of conditions. The center may be made up of a team of different specialists, sometimes also including scientists and researchers.


Specialist centers can support patients across your State and/or the United States, not just in their local area.

Do you know if there is a specialist center for your/your family member’s rare disease(s)?

- Yes. If you know the name of the specialist center, and the city, please specify it here: (1) ________________________________________________
- No (2)
- Don’t know (3)

Do you/your family member access a specialist center for your/their rare disease(s)?

- Yes (1)
- No (2)
- Don’t know (3)

If you/your family member does not access a specialist center, why not?

- There is not one (1)
- My insurance does not cover it (2)
- I live too far away (3)
- They are not accepting new patients (4)
- It is too long of a wait list to get in (5)
- Other. Please describe: (6) ________________________________________________
- Don't know (7)

How many different medical specialists (i.e., specialist doctors) do you/your family member see for your/their rare disease(s)?

- 1-2 (1)
- 3-4 (2)
- 5-6 (3)
- More than 6 (4)
- Don’t know (5)
- Not applicable (6)

What is the furthest distance traveled to access care for the rare disease(s)?

- Less than 10 miles (1)
- 10-29 miles (2)
- 30-59 miles (3)
- 60 or more miles (4)
- Internationally (5)
- Don't know (6)
- Not applicable (7)

Telehealth means using electronic information and telecommunications technologies like videoconferencing to provide long-distance health care. 


Do you use telehealth services to see medical specialists?

- Yes (1)
- No (2)
- Don’t know (3)
- Not applicable/I do not have access to the technology required for telehealth (4)

Would you be interested in telehealth/ehealth?

- Yes (1)
- No (2)
- Don’t know (3)
- Not applicable/I do not have access to the technology required for telehealth (4)

Care coordination refers to health care that is provided in a planned way that meets the needs and preferences of the patient. When care is coordinated well, the patient and his or her doctors, nurses, other health care providers, family, and other caregivers all know who is responsible for different parts of the patient’s care, and they communicate with each other so that everyone has the information they need.


A care coordinator is a trained professional who is responsible for coordinating health care services, for example between specialist clinics, hospital staff, GPs, allied health and non-government organizations.


Do you/your family member have a designated care coordinator?

- Yes (1)
- No (2)
- Don't know (3)

If you have used a care coordinator, rate on a scale from 1 to 10 how helpful it was, with 1 being not at all helpful to 10 being extremely helpful.

- 1 (4)
- 2 (5)
- 3 (6)
- 4 (7)
- 5 (8)
- 6 (9)
- 7 (10)
- 8 (11)
- 9 (12)
- 10 (13)

If you have used a care coordinator, what worked well and what did not?

________________________________________________________________

If you have not used a care coordinator, why not?

________________________________________________________________

During the past 12 months, did you/your family member visit the following providers for their rare disease(s) and were you/they satisfied with their care?

|  | Have you seen this type of provider? | | If yes, how satisfied were you with their care? | | | | | |
| --- | --- | --- | --- | --- | --- | --- | --- | --- |
|  | Yes (1) | No (2) | Very dissatisfied (1) | Dissatisfied (2) | Neutral (3) | Satisfied (4) | Very satisfied (5) | Don't know/Not applicable (6) |
| General practitioner (GP) (1) |  |  |  |  |  |  |  |  |
| Allied health (e.g., physical/physio therapist, occupational therapist, speech therapist) (2) |  |  |  |  |  |  |  |  |
| Mental health professional (e.g., social worker, psychologist, psychiatrist) (3) |  |  |  |  |  |  |  |  |
| Dentist (4) |  |  |  |  |  |  |  |  |
| Specialist doctor (e.g., geneticist, neurologist, allergist) (5) |  |  |  |  |  |  |  |  |
| Other (please describe) (6) |  |  |  |  |  |  |  |  |

Please describe you/your family member’s experiences and satisfaction with healthcare providers you/your family member visited during the past 12 months.

________________________________________________________________

________________________________________________________________

________________________________________________________________

________________________________________________________________

________________________________________________________________

End of Block: Specialist centers/care

Start of Block: Pediatrics

Pediatric health service is defined as the specialty of medical science concerned with the physical, mental, and social health of children from birth to young adulthood. Pediatric care encompasses a broad spectrum of health services ranging from preventive health care to the diagnosis and treatment of acute and chronic diseases.

Were you/your family member ever cared for by pediatric health services for your rare disease(s)?

- Yes (1)
- No (2)
- Don't know (3)

Overall, how satisfied or dissatisfied are you with the care you/your family member receive(d) from pediatric health services?

- Very dissatisfied (1)
- Dissatisfied (2)
- Neither satisfied nor dissatisfied (3)
- Satisfied (4)
- Very satisfied (5)
- Don't know (6)

Did you/your family member transition from pediatric to adult health services?

- Yes (1)
- No (2)
- Don't know (3)

What was the time period between your/your family member’s last visit to pediatric health services and the first visit to adult health services. Please enter the value in weeks (e.g., if one year, write 52 weeks).

________________________________________________________________

Was this time acceptable or unacceptable to you/your family member?

- Acceptable (1)
- Unacceptable (2)
- Don't know (3)

Did you/your family member experience any problems in the transition from pediatric to adult services?

- Yes. Please describe what the problems were related to: (1) ________________________________________________
- No (2)
- Don't know (3)

How would you compare the knowledge of your/your family member’s pediatric provider to your/your family member’s adult provider?

- They have similar expertise regarding the rare disease (1)
- The pediatric provider has more expertise regarding the rare disease than the adult provider (2)
- The adult provider has more expertise regarding the rare disease than the pediatric provider (3)
- Don’t know (4)

What other comments do you have about your/your family member’s experience using pediatric health services, including the transition from pediatric to adult care?

________________________________________________________________

End of Block: Pediatrics

Start of Block: Relocation

Thank you. Next, we have some questions about challenges you/your family member may have had getting care or treatment for the rare disease(s).

Did any of the following limit you/your family member’s ability to get medical or dental care or treatment for your/their rare disease?

|  | Never (1) | Sometimes (2) | About half the time (3) | Most of the time (4) | Almost always (5) | Don't know (6) |
| --- | --- | --- | --- | --- | --- | --- |
| Finances (for example, travel costs, cost of treatment/insurance) (1) |  |  |  |  |  |  |
| Travel distance (2) |  |  |  |  |  |  |
| Difficulty getting time off of work (3) |  |  |  |  |  |  |
| Lack of childcare (4) |  |  |  |  |  |  |
| Lack of or delay in referrals (5) |  |  |  |  |  |  |
| Not covered by insurance (6) |  |  |  |  |  |  |
| Other (please describe) (7) |  |  |  |  |  |  |

Have you/your family member needed to relocate in order to access treatment or clinical trials for their rare disease long-term?

|  | No, we have not relocated (1) | Temporarily (6 months or less) (2) | 7 months to 2 years (3) | 2 years or longer (4) | Permanently (5) | Don't know (6) |
| --- | --- | --- | --- | --- | --- | --- |
| In-state (1) |  |  |  |  |  |  |
| Out of state (2) |  |  |  |  |  |  |

If you/your family member relocated because of the rare disease, please describe why:

________________________________________________________________

End of Block: Relocation

Start of Block: Patient Satisfaction Questionnaire Short

Think about your overall experience with healthcare for your/your family member's rare disease(s) when responding to the following statements.

If you are a caregiver, please answer these questions in terms of how the person with the rare disease is feeling. If the person with a rare disease is under age 10, please answer based on your experience with them as a caregiver.

|  | Strongly disagree (1) | Disagree (2) | Neither agree nor disagree (3) | Agree (4) | Strongly agree (5) | Don't know (6) | Not applicable (7) |
| --- | --- | --- | --- | --- | --- | --- | --- |
| Healthcare providers are good about explaining the reason for medical tests (1) |  |  |  |  |  |  |  |
| I think my healthcare providers' office has everything needed to provide complete medical care (2) |  |  |  |  |  |  |  |
| The medical care I have been receiving is just about perfect (3) |  |  |  |  |  |  |  |
| Sometimes healthcare providers make me wonder if their diagnosis is correct (4) |  |  |  |  |  |  |  |
| I feel confident that I can get the medical care I need without being set back financially (5) |  |  |  |  |  |  |  |
| When I go for medical care, they are careful to check everything when treating and examining me (6) |  |  |  |  |  |  |  |
| I have to pay for more of my medical care than I can afford (7) |  |  |  |  |  |  |  |
| I have easy access to the medical specialists I need (8) |  |  |  |  |  |  |  |
| Where I get medical care, I have to wait too long for emergency treatment (9) |  |  |  |  |  |  |  |
| Healthcare providers act too businesslike and impersonal toward me (10) |  |  |  |  |  |  |  |
| My healthcare providers treat me in a very friendly and courteous manner (11) |  |  |  |  |  |  |  |
| Those who provide me medical care sometimes hurry too much when they treat me (12) |  |  |  |  |  |  |  |
| Healthcare providers sometimes ignore what I tell them (13) |  |  |  |  |  |  |  |
| I have some doubts about the ability of the healthcare providers who treat me (14) |  |  |  |  |  |  |  |
| Healthcare providers usually spend plenty of time with me (15) |  |  |  |  |  |  |  |
| I find it hard to get an appointment for medical care right away (16) |  |  |  |  |  |  |  |
| I am dissatisfied with some things about the medical care I receive (17) |  |  |  |  |  |  |  |
| I am able to get medical care whenever I need it (18) |  |  |  |  |  |  |  |

End of Block: Patient Satisfaction Questionnaire Short Form

Start of Block: PROMIS 29 profile – CHILDREN – THESE QUESTIONS ARE FOR PARENTS OF CHILDREN WITH RARE DISEASE(S)

Next, we will ask about your child's overall health and well-being.

|  | Excellent (1) | Very good (2) | Good (3) | Fair (4) | Poor (5) |
| --- | --- | --- | --- | --- | --- |
| In general, would you say your child's health is... (1) |  |  |  |  |  |

In the past 7 days...

|  | Never (1) | Almost never (2) | Sometimes (3) | Often (4) | Almost always (5) | Don't know (6) | Not applicable (7) |
| --- | --- | --- | --- | --- | --- | --- | --- |
| My child could do sports and exercise that other kids his/her age could do (1) |  |  |  |  |  |  |  |
| My child could get up from the floor (2) |  |  |  |  |  |  |  |
| My child could walk up stairs without holding onto anything (3) |  |  |  |  |  |  |  |
| My child has been physically able to do the activities he/she enjoys most (4) |  |  |  |  |  |  |  |

In the past 7 days...

|  | Never (1) | Almost never (2) | Sometimes (3) | Often (4) | Almost always (5) | Don't know (6) | Not applicable (7) |
| --- | --- | --- | --- | --- | --- | --- | --- |
| My child felt like something awful might happen (1) |  |  |  |  |  |  |  |
| My child felt nervous (2) |  |  |  |  |  |  |  |
| My child felt worried (3) |  |  |  |  |  |  |  |
| My child worried when he/she was at home (4) |  |  |  |  |  |  |  |

In the past 7 days...

|  | Never (1) | Almost never (2) | Sometimes (3) | Often (4) | Almost always (5) | Don't know (6) | Not applicable (7) |
| --- | --- | --- | --- | --- | --- | --- | --- |
| My child felt everything in his/her life went wrong (1) |  |  |  |  |  |  |  |
| My child felt lonely (2) |  |  |  |  |  |  |  |
| My child felt sad (3) |  |  |  |  |  |  |  |
| It was hard for my child to have fun (4) |  |  |  |  |  |  |  |

In the past 7 days...

|  | Never (1) | Almost never (2) | Sometimes (3) | Often (4) | Almost always (5) | Don't know (6) | Not applicable (7) |
| --- | --- | --- | --- | --- | --- | --- | --- |
| Being tired made it hard for my child to keep up with schoolwork (1) |  |  |  |  |  |  |  |
| My child got tired easily (2) |  |  |  |  |  |  |  |
| My child was too tired to do sports or exercise (3) |  |  |  |  |  |  |  |
| My child was too tired to enjoy the things he/she likes to do (4) |  |  |  |  |  |  |  |

In the past 7 days...

|  | Never (1) | Almost never (2) | Sometimes (3) | Often (4) | Almost always (5) | Don't know (6) | Not applicable (7) |
| --- | --- | --- | --- | --- | --- | --- | --- |
| My child felt accepted by other kids his/her age (1) |  |  |  |  |  |  |  |
| My child was able to count on his/her friends (2) |  |  |  |  |  |  |  |
| My child and his/her friends helped each other out (3) |  |  |  |  |  |  |  |
| Other kids wanted to be my child's friend (4) |  |  |  |  |  |  |  |

| Page Break |  |
| --- | --- |

 In the past 7 days...

|  | Never (1) | Almost never (2) | Sometimes (3) | Often (4) | Almost always (5) | Don't know (6) | Not applicable (7) |
| --- | --- | --- | --- | --- | --- | --- | --- |
| My child had trouble sleeping when he/she had pain (1) |  |  |  |  |  |  |  |
| It was hard for my child to pay attention when he/she had pain (2) |  |  |  |  |  |  |  |
| It was hard for my child to run when he/she had pain (3) |  |  |  |  |  |  |  |
| It was hard for my child to walk one block when he/she had pain (4) |  |  |  |  |  |  |  |

In the past 7 days...

|  | 1 No pain (1) | 2 (2) | 3 (3) | 4 (4) | 5 (5) | 6 (6) | 7 (7) | 8 (8) | 9 (9) | 10 Worst pain you can think of (10) | Don't know (11) |
| --- | --- | --- | --- | --- | --- | --- | --- | --- | --- | --- | --- |
| How bad was your child's pain on average? (1) |  |  |  |  |  |  |  |  |  |  |  |

| Page Break |  |
| --- | --- |

End of Block: PROMIS 29 profile - CHILDREN

Start of Block: PROMIS 29 profile Form – THESE QUESTIONS ARE FOR ADULTS WITH RARE DISEASE(S)

Next, we will ask about your/your family member’s overall health and well-being. If you are a caregiver, please answer these questions in terms of how the person with the rare disease is feeling.

|  | Excellent (1) | Very good (2) | Good (3) | Fair (4) | Poor (5) |
| --- | --- | --- | --- | --- | --- |
| In general, would you say your health is... (1) |  |  |  |  |  |

|  | Without any difficulty (1) | With a little difficulty (2) | With some difficulty (3) | With much difficulty (4) | Unable to do (5) | Don't know (6) |
| --- | --- | --- | --- | --- | --- | --- |
| Are you able to do chores such as vacuuming or yard work? (1) |  |  |  |  |  |  |
| Are you able to go up and down stairs at a normal pace? (2) |  |  |  |  |  |  |
| Are you able to go for a walk of at least 15 minutes? (3) |  |  |  |  |  |  |
| Are you able to run errands and shop? (4) |  |  |  |  |  |  |

In the past 7 days...

|  | Never (1) | Rarely (2) | Sometimes (3) | Often (4) | Always (5) | Don't know (6) |
| --- | --- | --- | --- | --- | --- | --- |
| I felt fearful (1) |  |  |  |  |  |  |
| I found it hard to focus on anything other than my anxiety (2) |  |  |  |  |  |  |
| My worries overwhelmed me (3) |  |  |  |  |  |  |
| I felt uneasy (4) |  |  |  |  |  |  |

In the past 7 days...

|  | Never (1) | Rarely (2) | Sometimes (3) | Often (4) | Always (5) | Don't know (6) |
| --- | --- | --- | --- | --- | --- | --- |
| I felt worthless (1) |  |  |  |  |  |  |
| I felt helpless (2) |  |  |  |  |  |  |
| I felt depressed (3) |  |  |  |  |  |  |
| I felt hopeless (4) |  |  |  |  |  |  |

In the past 7 days...

|  | Not at all (1) | A little bit (2) | Somewhat (3) | Quite a bit (4) | Very much (5) | Don't know (6) |
| --- | --- | --- | --- | --- | --- | --- |
| I feel fatigued (1) |  |  |  |  |  |  |
| I have trouble starting things because I am tired (2) |  |  |  |  |  |  |
| How run-down did you feel on average? (3) |  |  |  |  |  |  |
| How fatigued were you on average? (4) |  |  |  |  |  |  |

In the past 7 days...

|  | Very poor (1) | Poor (2) | Fair (3) | Good (4) | Very good (5) | Don't know (6) |
| --- | --- | --- | --- | --- | --- | --- |
| My sleep quality was (1) |  |  |  |  |  |  |

In the past 7 days...

|  | Not at all (1) | A little bit (2) | Somewhat (3) | Quite a bit (4) | Very much (5) | Don't know (6) |
| --- | --- | --- | --- | --- | --- | --- |
| My sleep was refreshing (1) |  |  |  |  |  |  |
| I had a problem with sleep (2) |  |  |  |  |  |  |
| I had difficulty falling asleep (3) |  |  |  |  |  |  |

| Page Break |  |
| --- | --- |

|  | Never (1) | Rarely (2) | Sometimes (3) | Often (4) | Always (5) | Don't know (6) |
| --- | --- | --- | --- | --- | --- | --- |
| I have trouble doing all of my regular leisure activities with others (1) |  |  |  |  |  |  |
| I have trouble doing all of the family activities that I want to do (2) |  |  |  |  |  |  |
| I have trouble doing all of my usual work (include work at home) (3) |  |  |  |  |  |  |
| I have trouble doing all of the activities with friends that I want to do (4) |  |  |  |  |  |  |

In the past 7 days...

|  | Not at all (1) | A little bit (2) | Somewhat (3) | Quite a bit (4) | Very much (5) | Don't know (6) |
| --- | --- | --- | --- | --- | --- | --- |
| How much did pain interfere with your day to day activities? (1) |  |  |  |  |  |  |
| How much did pain interfere with work around the home? (2) |  |  |  |  |  |  |
| How much did pain interfere with your ability to participate in social activities? (3) |  |  |  |  |  |  |
| How much did pain interfere with your household chores? (5) |  |  |  |  |  |  |

In the past 7 days...

|  | 1 No pain (1) | 2 (2) | 3 (3) | 4 (4) | 5 (5) | 6 (6) | 7 (7) | 8 (8) | 9 (9) | 10 Worst pain imaginable (10) | Don't know (11) |
| --- | --- | --- | --- | --- | --- | --- | --- | --- | --- | --- | --- |
| How would you rate your pain on average? (1) |  |  |  |  |  |  |  |  |  |  |  |

| Page Break |  |
| --- | --- |

End of Block: PROMIS 29 profile

Start of Block: Anticipated stigma scale

Rate the likelihood that you/your family member will encounter the experiences below in the future because of your rare disease(s). 


If you are a caregiver, please answer these questions in terms of how the person with the rare disease is feeling. If the person with a rare disease is under age 10, please answer based on your experience with them as a caregiver.

|  | Very unlikely (1) | Unlikely (2) | Neither unlikely nor likely (3) | Likely (4) | Very likely (5) | Don't know (6) |
| --- | --- | --- | --- | --- | --- | --- |
| A healthcare worker will blame you for not getting better (9) |  |  |  |  |  |  |
| A healthcare worker will be frustrated with you (10) |  |  |  |  |  |  |
| A healthcare worker will give you poor care (11) |  |  |  |  |  |  |
| A healthcare worker will think that you are a bad patient (12) |  |  |  |  |  |  |

End of Block: Anticipated stigma scale

Start of Block: Stigma scale

Indicate how much you agree or disagree with each of the statements about the way you have felt about your rare disease(s) lately...

If you are a caregiver, please answer these questions in terms of how the person with the rare disease is feeling. If the person with a rare disease is under age 10, please answer based on your experience with them as a caregiver.

|  | Never (1) | Rarely (2) | Sometimes (3) | Often (4) | Always (5) | Don't know (6) |
| --- | --- | --- | --- | --- | --- | --- |
| Because of my rare disorder(s), some people seemed uncomfortable with me. (1) |  |  |  |  |  |  |
| Because of my rare disorder(s), some people avoided me. (2) |  |  |  |  |  |  |
| Because of my rare disorder(s), I felt left out of things. (3) |  |  |  |  |  |  |
| Because of my rare disorder(s), people were unkind to me. (4) |  |  |  |  |  |  |
| Because of my rare disorder(s), people avoided looking at me. (5) |  |  |  |  |  |  |
| I felt embarrassed about my rare disorder(s). (6) |  |  |  |  |  |  |
| I felt embarrassed because of my physical limitations due to my rare disorder(s). (7) |  |  |  |  |  |  |
| Some people acted as though it was my fault I have my rare disorder(s). (8) |  |  |  |  |  |  |

End of Block: Stigma scale

Start of Block: Qualitative

Thank you! There are only a couple of questions left.
Is there anything that you would like to add about your/your family member’s experience with rare disease that will help the researchers better understand the challenges experienced by patients/families? If so, please share it here:

________________________________________________________________

________________________________________________________________

________________________________________________________________

________________________________________________________________

________________________________________________________________

Has the COVID-19 pandemic changed your ability to access care and services?

- Yes (1)
- No (2)
- Don't know (3)

If so, how has COVID-19 changed how you access care and services?

________________________________________________________________

________________________________________________________________

________________________________________________________________

________________________________________________________________

________________________________________________________________

If there were ONE SINGLE THING that could be provided by the healthcare system to improve your/your families experience with this rare disease, what would it be?

________________________________________________________________

________________________________________________________________

________________________________________________________________

________________________________________________________________

________________________________________________________________

This is the last question of the survey. Is there anything you would like to add?

________________________________________________________________

________________________________________________________________

________________________________________________________________

________________________________________________________________

________________________________________________________________

End of Block: Qualitative
