# Supplementary material for: Healthcare access, satisfaction, and health-related quality of life among children and adults with rare diseases
Source: Orphanet J Rare Dis. 2022 May 12;17:196. doi: 10.1186/s13023-022-02343-4 (PMC9096775; doi:10.1186/s13023-022-02343-4)
Supplement: Supplementary file 2 — Additional file 2: PROMIS and stigma scores for the two most frequent RDs in the sample. [file 13023_2022_2343_MOESM2_ESM.docx]

**Supplemental Material Table 1**

PROMIS and stigma scores among adults with RDs compared to population norms

Note. An anonymous reviewer requested analyses of specific RDs. With Cohen’s d = .5, alpha = .05, and power = .80, a sample of 34 was needed. Only the two most frequent RDs in our adult sample, Spinocerebellar Ataxia and Idiopathic Hypersomnia met these minimum sample size requirements. (We also excluded participants with more than one RDs for this particular analysis because the effects of multiple diseases could not be parsed.) * p < .01, ** p < .001, *** p < .0001.
